# *Toxoplasma gondii*, suicidal behaviour and suicide risk factors in US Veterans enrolled in mental health treatment

**DOI:** 10.14411/fp.2025.002

**Published:** 2025-01-09

**Authors:** Teodor T. Postolache, Erica Duncan, Poyu Yen, Eileen Potocki, Meghan Barnhart, Amanda Federline, Nicholas Massa, Aline Dagdag, Joshua Joseph, Abhishek Wadhawan, Colt D. Capan, Cameron Forton, Christopher A. Lowry, Heidi K. Ortmeyer, Lisa A. Brenner

**Affiliations:** 1 Veterans Health Administration, Rocky Mountain Mental Illness Research Education and Clinical Center (MIRECC), Veterans Integrated Service Network (VISN) 19, Aurora, CO, USA;; 2 Mental Illness Research, Education and Clinical Center (MIRECC), Veterans Integrated Service Network (VISN) 5, VA Capitol Health Care Network, Baltimore, MD, USA;; 3 Military and Veteran Microbiome: Consortium for Research and Education (MVM-CoRE), Aurora, CO, USA;; 4 Mood and Anxiety Program, Department of Psychiatry, University of Maryland School of Medicine, Baltimore, MD, USA;; 5 Atlanta Veterans Affairs Health Care System, Decatur, GA, USA;; 6 Emory University School of Medicine, Department of Psychiatry and Behavioral Sciences, Atlanta, GA, USA;; 7 VA Maryland Healthcare System, Baltimore VA Medical Center, Baltimore, MD, USA;; 8 Saint Elizabeths Hospital, Department of Psychiatry, Washington, DC, USA;; 9 Department for Neurodegenerative Sciences, Van Andel Research Institute, Grand Rapids, MI, USA;; 10 Department of Integrative Physiology and Center for Neuroscience, University of Colorado Boulder, Boulder, CO, USA;; 11 Baltimore VA Medical Center, Baltimore, MD, USA;; 12 Department of Medicine, University of Maryland School of Medicine, Baltimore, MD, USA;; 13 Departments of Physical Medicine and Rehabilitation, Psychiatry and Neurology, University of Colorado Anschutz Medical Campus, Aurora, CO, USA; 14 Share senior authorship

**Keywords:** depression, suicidality, impulsivity, sleep impairment, United States

## Abstract

Markers of chronic infection *Toxoplasma gondii* (Nicolle et Manceaux, 1908) have been associated with suicidal self-directed violence (SSDV). We present the results of the first study relating *T. gondii* IgG serology with suicide attempts and suicidal ideation in United States Veterans, known to have higher suicide rates than members of the general population. We also related *T. gondii* serology to SSDV risk factors, including valid and reliable measures of trait impulsivity, aggression, self-reported depression, and sleep disturbance. We recruited 407 Veterans enrolled at three Veterans Affairs Medical Centers with mean (*S.D.*) age = 45.6 (11.6) years; 304 men (74.7%); 203 with a history of SSDV and 204 with no history of any self-directed violence (SDV). Seropositivity and serointensity, categorised as high (top quartile) or low (lower three quartiles), were analysed in relationship to SSDV, suicidal ideation and clinical risk factors using age and gender-adjusted linear and logistic methods, after transformations and nonparametric tests when appropriate. Associations between seropositivity and SSDV and its risk factors were not significant in all groups. High serointensity, while not associated with SSDV or repeat suicide attempts, was positively associated with suicidal ideation, depression, impulsivity, and daytime dysfunction due to sleepiness (*p* < 0.05), but only in Veterans with a history of SSDV. In Veterans without a history of SDV, no associations were significant. These associations remained significant after adjustment for certain socioeconomic factors (i.e., income, homelessness, military rank). Including education in the model downgraded the statistical significance of suicidal ideation and depression to statistical trends, but the significance of associations with impulsivity and daytime dysfunction due to sleepiness remained. Major limitations include the cross-sectional design, overall low seropositivity within the sample, and potentially spurious results due to multiple comparisons. Thus, the results of this report need to be replicated in larger samples, ideally longitudinally.

Suicidal behaviour, also called suicidal self-directed violence (SSDV), including fatal (suicide) and non-fatal suicide attempts, is multi-factorially determined (Turecki and Brent 2016, Lutz et al. 2017). It is the result of the interplay between protective and aggravating factors, triggers and availability of means, often frustrating efforts to predict and manage risk (Joiner and Van Orden 2008). In the United States (US), suicide is one of the leading and unabating causes of death (Centers for Disease Control and Prevention 2023). US Veterans have higher age and gender-adjusted suicide rates as compared to those among members of the general population (US Department of Veterans Affairs 2023).

A wide range of biological factors associated with risk for suicidal behaviour have been explored (e.g., genetic, epigenetic, endocrine, neuroimmune, sleep, neurotransmitters, and neuroanatomical) (Mann 1998, Ernst et al. 2009, Mann et al. 2009, Dwivedi 2012, Pandey 2013, Oquendo et al. 2014, Van Heeringen and Mann 2014, Turecki and Brent 2016, Dwivedi 2018, Porras-Segovia et al. 2019). An expanding body of research implicates associations between infections and mental health conditions (Dalman et al. 2008, Benros et al. 2013, Burgdorf et al. 2019, Köhler-Forsberg et al. 2019, Nissen et al. 2019).

Additionally, evidence from multiple sources, including our own investigations, indicates associations between SSDV and various infections (Okusaga et al. 2011a, Lund-Sørensen et al. 2016, Gjervig Hansen et al. 2019, Nissen et al. 2019, Fallon et al. 2021), including chronic toxoplasmosis (Arling et al. 2009, Okusaga et al. 2011b, Pedersen et al. 2012, Zhang et al. 2012, Sutterland et al. 2019, Amouei et al. 2020, Soleymani et al. 2020).

*Toxoplasma gondii* (Nicolle et Manceaux, 1908) was discovered in 1908 and received its name in 1909 (Nicolle and Manceaux 1908, 1909). It is a widespread neurotropic protozoan parasite that sexually reproduces in the digestive tract of cats and produces abundant oocysts that are dispersed in the environment. Ingestion of oocysts by humans and any other warm-blooded animals, which play the role of intermediate hosts, is a common pathway involved in infection. Once ingested, the microorganism spreads from the intestine to other organs (primarily the brain and muscles), as tachyzoites (fast-growing forms). There, they transform under immune pressure into slow-growing forms (bradyzoites) confined to intracellular cysts within the brain and muscle. Ingestion of these tissue cysts results in the completion of the reproductive cycle of *T. gondii* in cats, while in humans and other intermediate hosts, it results in a second pathway of infection and dissemination from the intestine to the muscle and brain.

“Latent” chronic toxoplasmosis (henceforth designated as latent toxoplasmosis) is characterised by asymptomatic or oligosymptomatic persistence of cysts in host tissues, including in the brain. It is prevalent in 25–30% of the global population (Pappas et al. 2009) and 10–15% of the US population (Dubey and Jones 2008) – with higher prevalence in certain farming communities, such as among the Old Order Amish (Markon et al. 2020).

Reactivation of latent infection by *T. gondii* has been proposed to explain not only severe manifestations of toxoplasmosis among those with Acquired Immune Deficiency Syndrome (AIDS) and other conditions characterised by immunosuppression but may also contribute to intermittent exacerbations or reactivations of certain neurological, behavioural, affective, cognitive, and psychotic symptoms reported in individuals with latent toxoplasmosis (Grant et al. 1990, Meers et al. 2010).

Conversely, the host immune responses, innate and acquired, involved the parasite control, as well as *T. gondii*’s evasive capabilities in manipulating the immune system (Lima and Lodoen 2019, Frickel and Hunter 2021) – reciprocally interacting, could lead to an upregulated immune tone, intermittent reactivation and inflammation that could ultimately contribute to an increased risk of suicidal behaviour in affected hosts (Brundin et al. 2015).

## *Toxoplasma gondii* and suicidal behaviour

Our collaborative team first identified a positive link between *T. gondii*-specific IgG serointensity and previous suicide attempts among patients with mood disorders. (Arling et al. 2009). Building on this initial discovery, our team and other researchers have since validated the association between *T. gondii* serology and SSDV across a spectrum of psychiatric diagnoses. Specifically, we reported a positive association between *T. gondii* IgG serointensity and suicidal behaviour in younger persons with schizophrenia (Okusaga et al. 2011b), and in patients admitted for suicide attempts (Zhang et al. 2012).

We then confirmed, longitudinally, associations between *T. gondii* IgG seropositivity and stratified titres obtained from neonatal blood spots and subsequent SSDV in Danish mothers (Pedersen et al. 2012). In that study, the associations between *T. gondii* and subsequent suicide attempts were robust to adjustment for baseline mental illness (and even parental history of mental illness), previously linked with *T. gondii* infection, suggesting that mental illness does not fully mediate the relationship between *T. gondii* and SSDV. Three meta-analyses have confirmed the link between latent toxoplasmosis and suicidal behaviour (Sutterland et al. 2019, Amouei et al. 2020, Soleymani et al. 2020).

Repeated nonfatal SSDV (Hawton 1987) and SDV (Zahl and Hawton 2024) are associated with increased risk of subsequent fatal SSDV. Although a majority of studies have not found (Arling et al. 2009) or have not analysed the number of repeated past suicide attempts in relationship to *T. gondii* seropositivity and serointensity, the repeated attempt was positively associated with *T. gondii* seropositivity in individuals attending primary care health care clinics (Alvarado-Esquivel et al. 2021). The same study also reported significant associations between suicidal ideation and *T. gondii* seropositivity in a subsample of women and young adults (Alvarado-Esquivel et al. 2021). However, the link between *T. gondii* and suicidal ideation was not confirmed meta-analytically (Amouei et al. 2020). Moreover, in individuals with a substance use disorder, a negative rather than positive association between *T. gondii* and suicidal ideation was reported (Alvarado-Esquivel et al. 2015).

*Toxoplasma gondii* infection is associated with suicide risk factors, including clinical conditions, sleep disturbances, and impulsive and aggressive personality traits.

## Clinical conditions

Psychiatric conditions previously associated with an increased risk of SSDV and consistently linked with chronic *T. gondii* infection include schizophrenia (Torrey et al. 2007, 2012, Brown et al. 2005, Amminger et al. 2007, Mortensen et al. 2007, Niebuhr et al. 2008, Yolken et al. 2009, Sutterland et al. 2015) and bipolar disorder (Pearce et al. 2012, Hamdani et al. 2013, Sutterland et al. 2015, Oliveira et al. 2016, De Barros et al. 2017). In contrast, links between depression and *T. gondii* infection have been inconsistent.

Despite positive associations reported in several studies, such as among pregnant women (Groer et al. 2011), individuals with mental illness (Alvarado-Esquivel et al. 2016), and female Veterans (Duffy et al. 2015), a majority of studies failed to find significant associations between depression and chronic *T. gondii* infection (Pearce et al. 2012, Gale et al. 2014, 2016, Flegr and Hodný 2016, Alvarado-Esquivel et al. 2017). Certain studies that focused on identifying a state of depression rather than the trait (i.e., past history of depression) were more likely to find significant associations with *T. gondii* infection (Kamal et al. 2022, Söderholm et al. 2023). However, many of those studies did not find an association with depression (Gale et al. 2016).

## Sleep disturbances

Sleep impairment is prevalent among service personnel and persists even after discharge from the military (Bramoweth and Germain 2013). Sleep impairment is positively associated with SSDV, and with neuropsychiatric conditions that have been predictively associated with SSDV, such as depression (Nutt et al. 2008), traumatic brain injury (TBI), and posttraumatic stress disorder (PTSD) (Gilbert et al. 2015). Even after adjusting for these conditions, sleep impairment predicts suicidal ideation and behaviour in military personnel (being a stronger predictor than depression) (Ribeiro et al. 2012), and significantly elevates the risk of suicidal ideation and behaviour in Veterans (Pigeon et al. 2012, Bernert et al. 2015).

Sleep impairment is generally more rapidly correctable than other risk factors for SSDV (Porras-Segovia et al. 2019). *Toxoplasma gondii* possesses two genes encoding tyrosine hydroxylase (Gaskell et al. 2009), a key rate-limiting enzyme potentially involved in increased synthesis of the arousing/alerting neurotransmitter dopamine in latent toxoplasmosis. Additionally, *T. gondii* is also known to interfere with the inhibitory GABAergic activity (Brooks et al. 2015). Therefore, *T. gondii* infection has been hypothesised to be associated with insomnia.

While initial studies in nonclinical populations, including older adults (Meshreky et al. 2019) and healthy Old Order Amish (Ahmad et al. 2017, Corona et al. 2019) have found no association between insomnia and latent toxoplasmosis, a more recent study reported a positive association between high anti-*T*. *gondii* IgG antibody levels in individuals with insomnia compared to individuals without insomnia (Alvarado-Esquivel et al. 2022). Further, conflicting gender differences emerged, with reports of a stronger association in men, in particular in older men (Alvarado-Esquivel et al. 2022), contrasting with a report in students that found a significant association between *T. gondii* and insomnia in women but not in men (Khademvatan et al. 2016).

Experimental studies have found a marked decrease in slow-wave sleep and increased wakefulness in rodents chronically infected with *T. gondii*. These findings were attenuated by seven days of corticosteroid anti-inflammatory treatment, but not by antimicrobial treatment directly targeting replication (Dupont et al. 2021). A longer time since infection did not result in habituation of the sleep-wake abnormalities. In contrast, these abnormalities increased with time (Dupont et al. 2021). In sum, these features suggest that the alterations in sleep-wake in latent toxoplasmosis may be mediated by inflammation and that an ongoing priming effect could be contributory.

These increases in wakefulness, as well as locomotor activity (Webster et al. 1994), in rodents infected with *T. gondii* have been interpreted as potentially increasing the risk of predation and thus dissemination of the parasite, as an example of behavioural manipulation of the host by *T. gondii* (Dupont et al. 2021). In contrast, in the animal models of sleep in infections with acute (Fang et al. 1996, Tesoriero et al. 2019) and chronic pathogens (Olivadoti et al. 2011, Trammell and Toth 2014), infection is associated with decreased locomotion, sleepiness, and increased sleep duration – believed to be the effect of inflammation and its downstream cascades on brain sleep-wake structures (Dupont et al. 2021).

## Impulsive and aggressive personality traits

Latent toxoplasmosis has been associated with subclinical personality traits (Flegr et al. 2000, Fekadu et al. 2010, Lindová et al. 2010), personality disorders (Hinze-Selch et al. 2010), subtle neurological deficits (Havlíček et al. 2001), as well as a heightened risk of motor vehicle accidents (Flegr et al. 2002, Fekadu et al. 2010). Higher antibody titres were associated with motor vehicle accidents relative to subgroups with lower antibody titres. Variables associated with *T. gondii* serology have been implicated as precedent causes of car accidents and include prolonged reaction time (Flegr et al. 2024) and higher impulsivity and aggression (Cook et al. 2015, Coccaro et al. 2016).

In rodents, latent *T. gondii* infection reduces and even reverses the innate fear elicited by cat odour (Vyas et al. 2007). There is evidence that this behavioural manipulation does not occur only in rodents, but also in primates. Specifically, chimpanzees infected with *T. gondii* spend more time exploring leopard urine and thus risk being predated upon, rather than expressing immediate aversion to it, as noninfected chimpanzees do (Poirotte et al. 2016). Although some mixed findings have been noted, *T. gondii* infection has also been found to nonspecifically lower fear/anxiety and neophobia (Afonso et al. 2012). These behavioural changes in the host increase the chances of predation of intermediate hosts by felines, thus, aiding in the completion of the pathogen’s lifecycle.

In humans, trait impulsivity and aggression have been consistently associated with SSDV (Mann et al. 1999, Dumais et al. 2005) and have been proposed as endophenotypes of suicidal behaviour (Mann et al. 2009, Gould et al. 2017). We first reported associations of *T. gondii* infection with impulsivity and aggression in healthy adults with no personal or family history of mental illness, thus limiting the potential confounding effects of psychiatric conditions and their treatment (Cook et al. 2015). Additionally, *T. gondii* IgG seropositivity was significantly associated with higher impulsive sensation seeking among men and higher trait reactive aggression scores among women. We further confirmed the associations between *T. gondii* seropositivity and traits of both impulsivity and aggression in individuals with Intermittent Explosive Disorder, characterised by high levels of actual impulsive aggression (Coccaro et al. 2016).

No previous study has investigated the association between markers of latent toxoplasmosis and SSDV in US Veterans. We decided to focus on Veterans enrolled in mental health treatment to maximise risk for SSDV and to decrease heterogeneity of the sample. Thus, we primarily hypothesised a positive association of *T. gondii* IgG seropositivity and serointensity with SSDV in US Veterans, as previously reported in nonveterans. The secondary hypotheses tested in this study included positive associations of *T. gondii* IgG with suicidal ideation, repeat SSDV, and with other suicide risk factors previously reported in nonveterans, including history of prior depression, sleep impairment, impulsivity and aggression. As PTSD is comorbid with many psychiatric conditions and is linked with suicide risk (Sala-Hamrick et al. 2023), and particularly so in Veterans (Forehand et al. 2022), we adjusted *post hoc* the associations of *T. gondii* IgG with suicide risk factors for the presence of PTSD and the severity of PTSD symptoms.

## MATERIALS AND METHODS

### Overview of study design

This was a cross-sectional, observational, and convenience-based case-control study comparing Veterans in mental health treatment with a history of SSDV to Veterans without a history of any self-directed violence (SDV), suicidal or non-suicidal. The study was conducted at three VHA sites: one Mid-Atlantic, one Southeastern, and one in the Rocky Mountain Region. The Central VA IRB, as well as local regulatory bodies as needed, approved the protocol. All participants were required to sign an informed consent after being fully informed about the premises, requirements, risks, and procedures of the study. The participants’ capacity to sign informed consent was evaluated using a structured questionnaire.

### Recruitment methods

Several methods identified potential participants enrolled in mental health treatment: (1) electronic medical records (EMR) chart review and screening via the use of a partial HIPAA waiver for recruitment; (2) referrals of participants who met inclusion criteria and who were interested in participating; and (3) self-referral via an IRB-approved study flyer. Further, study staff used the VA EMR with a partial HIPAA waiver to identify patients who may qualify for the study based on relevant ICD-10 codes. Potential participants were sent a letter explaining the study and provided a means of contacting study staff to opt-in or opt-out of further screening for the study. If there was no response to the mailing, study staff made follow-up phone calls with a VA-approved, pre-written script. Prior to enrollment, study staff verbally administered a pre-screening form to all potential participants to determine initial eligibility. Participants were compensated for their participation in compliance with local VA policies.

### Inclusion criteria

Inclusion criteria were: 1) age 18–65 years; 2) history of at least one suicide attempt with a distinct intent to die (individuals with a history of SSDV) or no lifelong history of self-directed violence, either suicidal or non-suicidal (control group including individuals without history of SDV) – as confirmed by the Lifetime Suicide Attempt Self Injury Interview (L–SASI) (Linehan et al. 2006); and 3) a mental health diagnosis as confirmed by the administration of the Structured Clinical Interview for DSM–5, Research Version (SCID–5–RV).

### Exclusion criteria

Exclusion criteria were: 1) did not demonstrate an intact ability to sign informed consent, or did not meet L–SASI criteria for a history of SSDV (for attempters) or no history of SDV (for control participants); 2) had a positive lifetime history of stroke; 3) a history of heart attack or heart failure within the past six months; 4) hospitalisation for any medical conditions within the past 60 days; 5) requiring systemic corticosteroid or antibiotic treatment within the prior 60 days or any presence of medical condition known to affect immune system status (such as rheumatoid arthritis, ankylosing spondylitis, lupus and multiple sclerosis); 6) had a clinically significant memory deficit as indicated by a score of 19 or below on the Saint Louis University Mental Status (SLUMS) (Tariq et al. 2006) examination for detecting mild cognitive impairment and dementia – a scale that appears more sensitive than Mini-Mental Status Examination (MMSE) (Tariq et al. 2006), or a history of moderate- to severe-TBI as identified on The Ohio State University TBI–ID (Corrigan and Bogner 2007); 7) had a history of alcohol use disorder with active use over the past seven days; 8) same-day drug or alcohol use as identified on the University of Washington Risk Assessment Protocol-Revised (UWRAP) (Linehan et al. 2012); 9) failing to pass the Test of Memory Malingering (TOMM) (Tombaugh 1997, Teichner and Wagner 2004), as indicated by scoring below 25 on the first trial, or below 45 on the retention trial; 10) history of HIV infection by self-report and/or documentation in EMR record; and 11) pregnancy.

### Instruments for estimating the presence and severity of mental health and substance use disorder

The Structured Clinical Interview for DSM–5, Research Version (SCID–5–RV) (American Psychiatric Association 2013) is a validated semi-structured interview that was used to determine DSM–5 mental health and substance use diagnoses.

The Ohio State University TBI–ID (OSU TBI–ID) (Bogner and Corrigan 2009) was used to determine history of TBI and classify it as moderate/severe (exclusion), or mild (used for exploratory adjustments and stratifications).

The Posttraumatic Stress Disorder Checklist-5 (PCL–5) (Weathers 2013) is a psychometrically adequate self-report measure that assesses post-traumatic stress disorder (PTSD) symptoms in the past 30 days. Items correspond to DSM–5 diagnostic criteria for PTSD (American Psychiatric Association 2013). For PTSD we have used three measures – the SCID-derived diagnosis of current PTSD, or lifetime PTSD, or the continuous PCL–5 score with the PCL–5 categorised as positive (PCL > 33) and negative (PCL ≤ 33) (Bovin et al. 2016). PTSD was *a priori* designated as a potential confounder to be used for *post hoc* adjustments of significant results.

### Measures for dependent variables used in primary and secondary hypothesis testing

#### Measures of suicidal behaviour and ideation

The Lifetime Suicide Attempt Self Injury Interview (L–SASI) (Linehan et al. 2006) has psychometric properties that are well established, including good inter-rater reliability and adequate validity. It was used to define SSDV and SDV status.

The Beck Scale for Suicidal Ideation (BSS) (Beck et al. 1993) is the self-report version of the clinician-administered semi-structured Scale for Suicide Ideation (SSI). Response options ranged from 0–2 (lowest to highest severity) for each item on the scale, with a total scale ranging from 0–38. Items 20 and 21 refer to past suicide attempts and do not contribute to the overall score. Concurrent validity was previously reported as 0.90 (*p* <0.001) and internal reliability was similarly high (α = 0.93) (Beck et al. 1993).

#### Depression

The Beck Depression Inventory-II (BDI–II) is a well-validated and broadly used self-report measure of depression with excellent internal consistency (Beck et al. 1996) demonstrated in psychiatric outpatients, as well as high reliability, validity and internal consistency in primary care patients (Arnau et al. 2001). We have used the continuous total score and the categorised measure with a cut point of 14 and above to indicate the presence of clinical depression (Von Glischinski et al. 2019).

#### Sleep impairment

Sleep impairment was estimated with the Pittsburgh Sleep Quality Index (PSQI), an instrument with high internal reliability, test-retest reliability, sensitivity and specificity (Buysse et al. 1989). We used the total score and the categorised individual scores on six of the seven subcomponents were also analysed. The 18 items are grouped into several components, including sleep disturbance, prolonged sleep latency, daytime dysfunction due to sleepiness, impaired sleep duration, impaired sleep efficiency, and impaired sleep quality.

All variables were ranked with a minimum score = 0 (intact) and a maximum score = 3 (very impaired), with intermediate scores of 2 (mostly intact) and 3 (fairly impaired). We used binary transformations of each of the ranked sleep variables to a binary variable based on impairment being present (1) or absent (0). Specifically, measures of impaired sleep duration, sleep disturbance, impaired sleep efficiency, and impaired sleep quality were transformed to the binary value of 0 = impairment absent, (while the ranked values of 1, 2 and 3 were transformed to the binary value of 1 = impairment present. Exceptions were prolonged sleep latency (with ranked values 0, 1, 2 transformed to the binary value 0 – impairment absent, and the ranked value of 3 transformed to the binary value 1 – impairment present) and daytime dysfunction due to sleepiness transformed from the ranked values of 1, 2, 3 transformed to the binary value 1 – impairment present and ranked value of 0 assigned the binary value 0 – impairment absent, based on our prior unpublished calibration work.

#### Aggression

Aggression was measured with the Buss-Perry Aggression Questionnaire (Buss and Perry 1992) (BPAQ; with Verbal Aggression, Physical Aggression, Reactive Aggression, and Hostility as subscale scores). The BPAQ assesses a person’s tendency to act aggressively as a personality trait. It has excellent validity and reliability.

#### Impulsivity

Impulsivity was assessed using the Barratt Impulsiveness Scale Version 11 (BIS–11) (Patton et al. 1995). The BIS–11 is a 30-item questionnaire with good construct, convergent, and discriminant validity, with higher scores previously reported in individuals with a history of SSDV compared to those of psychiatric controls (Keilp et al. 2001).

#### Data collection and data entry

Data were collected by experienced staff members who completed training in administering research interviews. The reliability was crosschecked across sites. The Measures Training Team included two clinical research psychologists. They used video-conferencing to provide teaching practice and to verify the accurate and consistent administration of measures prior to meeting with study participants. They were also available for ongoing consultation and training during data collection to prevent rater drift, in addition to assessing inter-rater reliability.

Senior members of the research team and the Research Study Coordinator worked with the data manager to ensure the integrity of the data. To minimise data entry errors (or to identify “problem areas”), 25% of the data was entered twice and then compared side-by-side to assess intra- and inter-data entry reliability.

#### Collection of blood samples

A phlebotomy of a peripheral vein to obtain approximately 45 cc of blood was performed at least two hours after waking up and at least two hours after food consumption. Blood was drawn and processed by certified technicians in VA-approved laboratories. After centrifugation and separation of plasma and serum, processed separately, aliquots were stored in −80 °C freezers at the three primary locations. Coded and blinded specimens were sent to the Van Andel Research Institute (Lena Brundin lab) for analysis.

#### Laboratory measurements

*Toxoplasma gondii* IgG serointensity was measured using a Tecan Infinite M200 Pro plate reader. Samples were loaded into IBL America *T. gondii* IgG ELISA plates, per manufacturer protocol, and plated in duplicate (Immuno-Biological Laboratories Inc., Minneapolis, MN, USA). Calculated serointensity values were generated by way of a four Parameter Logistic Curve Calculator created by AAT Bioquest (“Quest Graph^™^ Four Parameter Logistic (4PL) Curve Calculator.” AAT Bioquest, Inc, 07 Dec. 2020, https://www.aatbio.com/tools/four-parameter-logistic-4pl-curve-regression-online-calculator). Samples that measured above a +/− 20% “grey area” from the value of the lowest calibrator were determined to be positive, while samples that fell below were determined to be negative. Samples that fell within the “grey area” were tested an additional time, if necessary. Five (1.23%) samples tested neutral and when re-tested, two were confirmed negative (0.49%) and the remaining three (0.74%) remained neutral or inconclusive. All diluted standards fell within the acceptable range as outlined by the manufacturer’s protocol.

#### Quartiles and dichotomised *Toxoplasma gondii* IgG

The *T. gondii* IgG serointensity was very skewed, which made it impossible to transform the distribution to normal (even with the loglog transformation). Thus, for statistical analysis, we dichotomised serointensity according to quartiles and analysed as the top quartile (TQ-Tg-IgG) versus the bottom three quartiles of serointensity, as previously reported (Mortensen et al. 2007, Pedersen et al. 2011, 2012).

### Statistical analysis

Initial analyses assessed the proportions of TQ-Tg-IgG participants in SSDV and SDV using chi-square tests. Demographic characteristics (age, gender, diagnosis), education, and VA site were summarised by TQ-Tg-IgG positivity and SSDV status. When log and loglog transformations were determined to improve distributions, we used the continuous variables, and when they did not, we categorised into quartiles – specifically top quartile and bottom three quartiles. A hierarchical mixed model was used to evaluate the impact of study site on the association between (TQ-Tg-IgG), suicidal ideation, and suicide risk factors.

The likelihood ratio tests compared the mixed model with the simpler regression models, and, as no significant predictive improvement was identified, simpler logistic regressions and one-way between-subjects ANCOVAs with adjustment for age, were used. Subsequent adjustments used logistic regressions and two-way ANCOVAs with subsequent adjustments for age and sex, age and education, and *post hoc* age and social economical confounders. We checked the assumptions of all models we fit, such as the GLM (e.g., logistic vs. probit), functional form (e.g., linear vs. nonlinear), by comparing sample and model-based statistics, and examining residual plots (Cook and Weisberg 2009).

Key variables that we always primarily adjusted for were age (mainly because of the irreversible caseness for both seropositivity as well as “attempter status”, both variables being positively associated with age and gender (because of striking gender differences in the association between personality factors and *T. gondii* seropositivity with personality traits that have been related to suicidal behaviour. These have been explained either by direct gender-specific predictive associations between latent toxoplasmosis and personality traits (Lindová et al. 2006) or, alternatively, via general gender-specific reactions to stressors, which include *T. gondii* infection (Lindová et al. 2010). *Post hoc*, successively, we adjusted for individual socioeconomic factors associated with the SSDV variables (income, education, rank, and homelessness) that were entered successively in exploratory regression models.

Exploratorily, we also successively adjusted *post hoc* the significant findings for certain medications and clinical conditions. To determine significant differences between individuals with a history of SSDV and individuals without history of SDV, we used the Fisher’s exact test to calculate the *p*-value, if the diagnosis or medication group had an *N* of 20 cases or less. If *N* was above 20, Pearson’s chi-squared test was used.

Finally, we adjusted the significant associations for PTSD measures. These included current and lifetime PTSD, and a categorised PCL–5 (cutoff > 33 for positive PCL–5 and ≤ 33 for negative PCL–5 [Bovin et al. 2016]), using multivariable logistic regressions and one-way between subjects ANCOVAs, with age included in the model.

## RESULTS

The sample included 203 (49.9%) Veterans with a history of SSDV and 204 (50.1%) without a history of SDV. In Atlanta, 179 Veterans were consented for screening. Of these, 73 screened out, and one withdrew. Consequently, 105 (58.7%) Veterans were enrolled and completed the procedures. In Baltimore, 152 Veterans were consented for screening, with 32 screening out and two withdrawing, with 118 (77.6%) who were enrolled and completed the procedures. In Denver, 226 Veterans were consented for screening, with 41 screening out and one withdrawing, and 184 Veterans (81.4%) enrolled in the study who completed the procedures. Thus, 73% of the Veterans passed the screening and agreed to participate in the study. Among the individuals with a history of SSDV, 116 (58.0%) had more than one attempt (i.e., >1 attempt) and 84 had a single attempt (42.0%). The percentages for multiple versus single attempts were based on the availability of data, limited to two hundred individuals. The description of the sample by history of attempt is presented in Table 2 (Demographic and socioeconomic information), Table 3 (Military history), Table 4 (Mental health and substance use diagnosis), and Table 5 (Psychotropic treatment).

Specific diagnostic and treatment distribution among those with a history of SSDV versus those without a history of SDV are presented in Table 4 and Table 5. The stratification by high versus low *Toxoplasma gondii* serointensity of the mental health and substance use disorders is presented in Table 6, and of the groups of medication is presented in Table 7.

In demographics analyses, the individuals with a history of SSDV had a higher proportion of women and lower proportion of men relative to individuals with no history of SDV (*p* = 0.07). There was also a trending association between college graduation and attempter status, such that the individuals with a history of SSDV had a trending lower proportion of college graduates relatively to individuals with no history of SDV (*p* = 0.07). Among those with a history of SSDV there was a greater proportion of individuals with a yearly income of ≥ $35,000 (*p* = 0.003), who were unemployed (*p* = 0.049), and homeless (*p* = 0.003) relative to individuals with no history of SDV. No other demographic variables were different between individuals with a history of SSDV and those without history of SDV (Table 2).

### Primary hypothesis testing

Only 25 Veterans were *T. gondii* IgG seropositive (6.14%). No significant associations between *T. gondii* seropositivity and any suicide attempt or suicide risk variables were identified (Table 8). The association between *T. gondii* seropositivity and suicide attempt status was not significant when unadjusted (OR 0.933, 95% CI [0.415, 2.097], *p* = 0.866) or adjusted for age (OR = 0.934, 95% CI [0.414, 2.106], *p* = 0.870).

The top quartile *T. gondii* IgG serointensity (TQ–Tg– IgG) was not statistically associated with SSDV (age-adjusted OR 1.133, 95% CI [0.729, 1.759], *p* = 0.579). On further exploration, this association was nonsignificant after multiple successive adjustments for confounders, including gender (adjusted OR 1.105, 95% CI [0.710, 1.720], *p* = 0.659). The results remained non-significant after successive adjustments for diagnostic categories, that were significantly different between individuals with a history of SSDV and individuals without a history of SDV.

### Suicidal ideation

In individuals with a history of SSDV, the BSS score, rank transformed, was significantly positively associated with TQ–Tg–IgG after adjustment for age (*F*_(1,197)_ = 5.53, *p* = 0.020, *r*^*2*^ = 0.039), as well as age and gender (*F*_(1,195)_ = 4.98, *p* = 0.027, *r*^*2*^ = 0.048) in an ANCOVA model. Despite the statistical significance, the elevation in BSS in the TQ–Tg–IgG group relative to bottom three quartiles group was rather small (only 6.7% higher).

This association in individuals with a history of SSDV remained significant with sequential *post hoc* adjustments for homelessness and age (*F*_(1,195)_ = 7.91, *p* = 0.005, *r*^*2*^ = 0.057), adjustments for military rank and age (*F*_(1,195)_ = 5.18, *p* = 0.024, *r*^*2*^ = 0.055), and income and age (*F*_(1,195)_ = 5.28, *p* = 0.023, *r*^*2*^ = 0.048). The significance decreased to trending after adjusting for education and age (*F*_(1,195)_ = 2.89, *p* = 0.091).

In individuals without history of SDV, the association of TQ–Tg–IgG with BSS scores was not significant (Table 9). In the total sample, the association was positively trending when adjusting for age (*F*_(1,399)_ = 3.377, *p* = 0.067) and also for age and gender (*F*_(1,397)_ = 3.081, *p* = 0.080) (Table 9).

### Repeated suicide attempts

In individuals with a history of SSDV, there was no association between TQ–Tg–IgG and repeated attempt status (one versus multiple attempts) when adjusted for age and gender (OR 0.842, 95% CI [0.445, 1.595], *p* = 0.598).

### Risk factors and endophenotypes in individuals with a history of SSDV

#### Depression

TQ–Tg–IgG was significantly positively associated with the log of total depression scores on BDI-II in individuals with a history of SSDV (Table 9) when adjusted for age: (*F*_(1,184)_ = 5.45, *p* = 0.021, *r*^*2*^ = 0.035) but when gender was added to the model, the statistical significance was reduced to a trend (*F*_(1,182)_ = 3.14, *p* = 0.078).

In individuals with a history of SSDV, TQ–Tg–IgG was also significantly positively associated with categorical depression – defined as BDI-II score ≥14 – with adjustment for age (OR 2.016, 95% CI [1.001, 4.059], *p* = 0.050) and age and gender (OR 2.037, 95% CI [1.007, 4.120], *p* = 0.048) (Table 9). *Post hoc* additional sequential adjustment was exploratory, unsupported by adequate statistical power. Nevertheless, when accounting for personal indicators of socioeconomic status, the association between TQ–Tg–IgG and categorical depression via BDI-II criteria was robust to adjustment for military rank and age (OR 2.054, 95% CI [1.016, 4.150], *p* = 0.045), adjustment for homelessness and age (OR 2.102, 95% CI [1.033, 4.279], *p* = 0.040), and adjustment for income and age (OR 2.019, 95% CI [1.001, 4.069], *p* = 0.050), but after adjustment for education the statistical significance decreased to a statistical trend (OR 1.980, 95% CI [0.981, 3.996], *p* = 0.057).

There were no significant associations between continuous and categorical BDI-II and TQ–Tg–IgG in individuals with no history of SDV, or in the entire sample (Table 9).

#### Impulsivity

In individuals with a history of SSDV, the top quartile BIS–11 score was significantly positively associated with TQ–Tg–IgG after adjustment for age (OR 1.998, 95% CI [1.056, 3.779], *p* = 0.033) and for age and gender (OR 2.020, 95% CI [1.064, 3.835], *p* = 0.032) (Table 9). The association was not significant in individuals without a history of SDV (Table 9).

*Post hoc* additional sequential adjustment was unsupported by adequate statistical power. Even so, the statistical significance of the association between BIS–11 and TQ– Tg–IgG resisted adjustment for individual indicators of socioeconomic status – income, homelessness, education, and military rank (income and age adjustment (OR 2.050, 95% CI [1.079, 3.893], *p* = 0.028); education and age adjustment (OR 1.921, 95% CI [1.010, 3.656], *p* = 0.047); military rank and age adjustment (OR 2.058, (1.080, 3.919], *p* = 0.028); and homelessness and age adjustment (OR 2.064, 95% CI [1.089, 3.986], *p* = 0.027).

There were no significant associations between impulsivity and TQ–Tg–IgG in individuals with no history of SDV, or in the entire sample.

#### Aggression

There were no significant associations between serointensity based on grouping categories, and the BPAQ scores of aggression (total score, physical aggression, verbal aggression, hostility, and anger scores) the entire sample, and separately for individuals with a history of SSDV and individuals without SDV (Table 9).

#### Sleep

Most Veterans with a history of SSDV (*n* = 170, 84.2%) and Veterans without a history of SDV (*n* = 150, 74.3%) reported daytime dysfunction due to sleepiness. The impaired overall sleep quality, sleep duration, sleep efficiency, prolonged sleep latency, and the total PSQI score were not significantly associated with TQ–Tg–IgG in the entire sample, and separately in individuals with SSDV and those without SDV (Table 9).

Among all sleep quality variables, TQ–Tg–IgG was only significantly positively associated with the daytime dysfunction due to sleepiness and only in individuals with a history of SSDV (Table 9). This association resisted adjustment for age (OR 2.850, 95% CI [1.059, 7.672], *p* = 0.038), and for age and gender (OR 2.849, 95% CI [1.058, 7.675], *p* = 0.038).

Additionally, the association between TQ–Tg–IgG and daytime dysfunction due to sleepiness remained significant after *post hoc* adjustments for certain salient measures of socioeconomic status – including income (OR 2.809, 95% CI [1.040, 7.586], *p* = 0.042); education (OR 2.811, 95% CI [1.042, 7.585], *p* = 0.041); homelessness (OR 2.837 95% CI [1.043, 7.714], *p* = 0.041); and military rank (OR 2.938, 95% CI [1.085, 7.956], *p* = 0.034).

#### Adjustment of significant findings for PTSD

Considering the significant differences between the SSDV Positive and SDV Negative groups in PTSD rates (Table 4), military service history, and military sexual trauma (Table 3), as specified *a priori* by the study protocol, we adjusted, *post hoc*, all the significant reported above for PTSD. These results remained significant after adjustment for all three measures for PTSD (Table 10).

## DISCUSSION

In the first study on latent toxoplasmosis and SSDV and suicide risk factors in US Veterans, we have not confirmed our specific hypothesis that seropositivity and having elevated *Toxoplasma gondii* IgG are associated with a history of suicide attempt. We also did not confirm our hypothesised positive association between *T. gondii* IgG serology and history of multiple suicide attempts (originating from the consideration for a potential intermittent reactivation of *T. gondii*). We did not identify data in support of the relationship between *T. gondii* seropositivity and any suicide attempt/risk variables, potentially as a result of low seropositivity in the studied sample. However, in Veterans with a history of SSDV only, we confirmed secondarily hypothesised associations between high IgG serointensity and suicidal ideation, impulsivity, depression, and daytime dysfunction due to sleepiness. Specifically, there was a >100% increase in odds ratios of having high impulsivity, depression, and daytime dysfunction due to sleepiness in Veterans in the TQ–Tg–IgG group.

The majority (78.3%) of Veterans in the TQ–Tg–IgG group were seronegative. Sub-positivity thresholds of IgG antibodies against a pathogen refer to levels that are detectable but below the established threshold for a positive result. Cross-reactivity is a complex process that involves interplay among microbial targets, self-antigens, and components of the immune system (Frank 2002), and a relatively common cause of sub-positive significant associations. In a recent systematic review and meta-analysis, cross-reactivity to other microbes was evaluated in human studies using serum that tested positive for various pathogens. These included viruses such as herpes simplex virus, cytomegalovirus, and rubella virus, helminths, including species of *Schistosoma*, *Fasciola* and *Echinococcus*, and protists, like *Trypanosoma cruzi*, species of *Leishmania*, and *Plasmodium*; the overall cross-reactivity of *T. gondii* with other microbes was 18.6% (Huertas-López et al. 2024). Of broader relevance, infection targeting antibodies cross-react with self-antigens with or without autoimmunity, in part via molecular mimicry (Cusick et al. 2012, Rojas et al. 2018). Thus cross-reactive antibodies may be the consequence of immune activation and induction of autoimmunity in toxoplasmosis (Trier and Houen 2023), with nonspecific immune, allergic, musculoskeletal, and metabolic disorders having been reported to be associated with latent toxoplasmosis (Flegr and Escudero 2016).

Elevated levels of sub-positive antibodies can result from several sources, such as cross-reactivity caused by shared epitopes, low-grade autoimmune responses (particularly when the pathogen imitates self-antigens), and non-specific binding or background noise in the test. The assay’s sensitivity is a key factor, as highly sensitive tests can detect very small amounts of specific antibodies that cross-react. In contrast, a high specificity of the test has a lower likelihood of cross-reactivity, even at lower concentrations. The timing of infection is critical with initial responses having lower cross-reactivity, as compared to sustained sub-positive levels. The generation of cross-reactive antibodies is also modified by genetic makeup, age, and general health.

With regards to *T. gondii*, it is possible for antibodies against other pathogens or antigens from the host to mistakenly react with *T. gondii* antigens, resulting in sub-positive or positive serology. While sub-positivity threshold IgG antibodies against a pathogen could potentially be cross-reactive autoimmune antibodies, this is not necessarily more likely than other possibilities. The specificity of the antibodies, the sensitivity of the assay, and the timing of the immune response (long durations since infection without reactivations could results in lowering serointensity, potentially – in some individuals below the seropositivity threshold, could all play a role in determining the potential for cross-reactivity and autoimmunity. These low titre antibodies could also represent a simple marker of a chronic infection – being known that longer infection with a cumulative effect of exposure is more often associated with personality alterations – than recent infections known in general to have a higher antibody titre (Flegr et al. 1996).

Because the TQ–Tg–IgG was significantly associated with SSDV risk factors but seropositivity was not, one could speculate that it is likely that the subthreshold, possibly cross-reacting antibodies in the seronegative subgroup within the TQ–Tg–IgG group are responsible for the significant results in the Veterans with SSDV. However, if the individuals with subthreshold serointensity (seronegative) within the TQ–Tg–IgG group drove the main significant findings in the TQ–Tg–IgG group, we would expect to find significant elevations in measures of suicide risk factors in the seronegative versus seropositive subgroup within the TQ–Tg–IgG. However, *post hoc*, there were no such differences, with the exception of a statistically trending difference with higher log BDI-II scores in the seronegative versus seropositive group within the TQ–Tg–IgG.

We did not find any significant differences in measures of suicide risk factors between the seropositive and seronegative subgroups within the TQ–Tg–IgG in the entire sample. There was a negatively trending association with log of BDI-II score total when unadjusted and adjusted for age (Table 11). There were no significant or trending associations with stratification by SSDV/SDV status. Thus, it is more likely that increased statistical power of the TQ– Tg–IgG analysis as compared to seropositivity analysis is the main underlying factor for significant findings in the former but not in the latter.

Some of our findings, such as an increase in daytime dysfunction due to sleepiness, are prone to be interpreted as a consequence of *T. gondii* being associated with general poor health, including conditions that were previously reported as being associated with latent toxoplasmosis (Flegr and Escudero 2016) and that may be a consequence, at least in part, of immune dysregulation possibly including autoimmune mechanisms.

Considering generalisability and relevance to Veterans enrolled in mental health treatment, we have used a real-life sample with relatively broad inclusion criteria. It is possible that the high heterogeneity of the sample in terms of substance abuse, demographic, clinical, and trauma history may have contributed to negative primary results and secondary associations, which, although significant, would not withstand adjustments for multiple comparisons. However, there are also strengths in the results, such as the significant associations being consistent with prior literature and being present only in Veterans who attempted suicide. Furthermore, all the main significant results in Veterans who had a history of a suicide attempt resisted adjustment for PTSD (Table 10) and yielded robust post-adjustment outcomes for three different PTSD measures (current, lifetime and PCL-based), making it unlikely as an artifact.

In several *post hoc* exploratory adjustments, given that the direction of the hypothesis was both predefined and already confirmed in the unadjusted association, a case could be made for the adequacy of one-tailed tests, especially considering the limitations in statistical power. If one-tailed tests had been used, all results of the study would have been robust to adjustment for all socioeconomic confounders. However, we refrained from implementing that approach as the study project protocol explicitly *a priori* prescribed two-tailed testing for all analyses.

A prior diagnosis of depression (trait) has generally been shown to insignificantly relate to *T. gondii* serology, in contrast to prior significant positive associations with the severity of the depressed state. Indeed, our identified links between *T. gondii* IgG and BDI-II continuous scores (statistical trend after adjustment) and categorical threshold-defined depression (statistically significant), are consistent with previously reported associations between current (state rather than trait) severity of symptoms of depression and *T. gondii* serointensity in pregnant women (Groer et al. 2011), female Veterans (Duffy et al. 2015), and Amish participants (Wadhawan et al. 2017). Similarly, a large study representative of the Finnish general population did identify a significant relationship between *T. gondii* seropositivity and a higher current BDI-II score (Suvisaari et al. 2017), but no such significant association with a history of major depression, dysthymia, or any depressive disorder within the past 12 months (Suvisaari et al. 2017). As in prior literature, we found no association between *T. gondii* IgG and a lifelong diagnosis of major depression, using SCID–5–RV.

### Daytime dysfunction due to sleepiness

Daytime dysfunction due to sleepiness is often the consequence of chronic sleep deficit (Cohen et al. 2010) and of a number of conditions that affect circadian and sleep dysregulation (Slater and Steier 2012). Excessive daytime sleepiness is common in US Veterans who participated in the Operations Enduring Freedom and Iraqi Freedom (Plumb et al. 2014) and has been identified as a predictor for suicidal ideation in a French cohort of outpatients with treatment-resistant depression (Maruani et al. 2023), and, furthermore, predicts suicide risk in adults admitted to an inpatient psychiatric unit (Shepard et al. 2023). It does not appear that the daytime dysfunction due to sleepiness is simply a marker of sleep disturbance as, even after adjustment for other sleep-related variables, it emerged as a significant risk factor for suicidal ideation and suicide planning in a large longitudinal study of adolescents in China (Liu et al. 2019). Although we have used subjective reporting rather than objectively measuring or estimating sleep, subjective ratings were reported to manifest stronger associations with anxiety and depression than the objective measures (Gould et al. 2018a,b). Our results are inconsistent with our two previous studies in the Old Order Amish. In the first one, we found a significant unadjusted relationship between *T. gondii* seropositivity and a clinically validated measure for daytime sleepiness, the Epworth Sleepiness Scale, but after demographic adjustments, statistical significance was lost (Ahmad et al. 2017). The second project found daytime sleepiness to be significantly reduced in *T. gondii* seropositives (Corona et al. 2019). These differences could be a result of participant characteristics.

In this project, we have recruited Veterans currently in treatment for multiple mental health conditions that could impair sleep and magnify *T. gondii’s* contribution to sleepiness. While in the previous studies of Old Order Amish, most participants were mostly psychiatrically healthy individuals. Additionally, the majority of the Amish participants lead a healthy lifestyle without alcohol or drugs, are regularly active as farmers, and have limited contact with technology (e.g short wavelength light emitting TV, smart phones, computers and tablets) (Lee et al. 2020) that is known to cause sleep disturbances. Differential exposure to past psychological trauma could also explain potential additive or synergistic effects magnifying the effects of chronic infection on sleep impairment and daytime dysfunction due to sleepiness in Veterans (Alter et al. 2021).

### Impulsivity

In Veterans with a history of SSDV we confirmed the expected significant association between TQ–Tg–IgG with higher impulsivity. This is consistent with our two prior reports – one in healthy individuals (Cook et al. 2015) and one in patients with mental illness. However, in contrast our prior report in patients with intermittent explosive disorder (Coccaro et al. 2016), the significant impulsivity link was not parallelled by a significant association of aggressive traits with TQ–Tg–IgG.

### Potential mechanisms

The prolonged presence of *T. gondii* in the brain causes permanent alterations in the regulation of numerous cellular functions and signaling pathways, including apoptosis, oxidative stress, and inflammation. This is accomplished in part by manipulating the overall expression of certain microRNAs, contributing to the parasite’s capacity to evade the immune system (Lima and Lodoen 2019, Frickel and Hunter 2021), sustain an infection for an extended period of time, and either promote or reduce harm to tissues (Hou et al. 2022). The parasites are able to elicit direct alterations in the infected cells by modifying dopamine metabolism, functionally silencing neurons, slowing autophagy and apoptosis, and by altering synaptic plasticity and neuronal connectivity (Parlog et al. 2015).

To minimise detection and targeting by the immune system, the parasite adopts a strategy of seclusion within cystic formations located within glial cells and neurons, keeping itself separate from lytic organelles and inhibiting autophagy. Cysts harbouring *T. gondii* predominantly accumulate within brain regions associated with the elicitation of fear responses, namely the amygdala, as well as regions involved in the regulation of fear, such as the prefrontal cortex (Vyas et al. 2007, Berenreiterová et al. 2011, McConkey et al. 2013). Specifically, the presence of persistent infection with *T. gondii* has the ability to influence behaviour by causing a decrease in fear response and an increase in risk-taking behaviour. This effect may occur through the process of dendritic retraction in the basolateral amygdala (Mitra et al. 2013).

The bradyzoite biology and its reactivation could be responsible for intermittent affective and behavioural dysregulation and is strongly linked with the immune response and activation of inflammatory and anti-inflammatory pathways during chronic infection (Zhao and Ewald 2020). The cell-autonomous, innate, and innate-conducted acquired immune activation are the most important components of defence against *T. gondii*, by limiting reactivation in latent toxoplasmosis. *T. gondii*-specific IgG antibodies and elevated serum cytokines (both proinflammatory and anti-inflammatory) serve as biomarkers of chronic infection and its course (Zhao and Ewald 2020).

It is now well known that many infections could induce and perpetuate the production of autoantibodies in the host which sometimes result in autoimmune-like conditions (Rivera-Correa and Rodriguez 2018, Trier and Houen 2023). Indeed, there is evidence that *T. gondii* infection triggers or modulates autoimmune disease and *T. gondii* targeting antibodies may cross-react with host tissues. For instance, *T. gondii* positivity is associated with systemic lupus erythematosus (Li et al. 2024), juvenile idiopathic arthritis (diagnosis and severity) (Salem et al. 2023), and type 1 diabetes (Asgari et al. 2021). Moreover, pathogens and autoimmunity in the central nervous system are connected via molecular mimicry (Münz et al. 2009). For example, anti-*N*-methyl-*D*-aspartate (anti-NMDA) receptor encephalitis is an autoimmune disorder triggered by autoantibodies against the NR1 subunit of the NMDA receptor, which could be induced by chronic *T. gondii* infection, more precisely by tissue cyst formation (Li et al. 2018).

Furthermore, individuals with anti-NMDA receptor encephalitis have an increased incidence of suicidal ideation and behaviour (Zhang et al. 2017). Indeed, patients with autoimmune disease have an increased prevalence of *T. gondii* antibodies (Shapira et al. 2012). Furthermore, autoantibodies, such as antinuclear antibodies, have been generated in *T. gondii* infection and could mediate certain clinical manifestations in autoimmune disease (Radon et al. 2003). Multiple mechanisms have been implicated in the onset of autoimmunity in the context of infection, including bystander activation (Shim et al. 2022), defective self-tolerance (Theofilopoulos et al. 2017), epitope spreading (Cornaby et al. 2015), and molecular mimicry (Cusick et al. 2012, Rojas et al. 2018).

Even before any infection or autoimmunity, the existence of natural antibodies targeting both microbes and self-antigens has been reported. They have been described as a first line of defense against pathogens and are known to have a protective effect in both infections and autoimmune diseases (Rivera-Correa and Rodriguez 2018).

The increased impulsivity, an endophenotype of suicidal behaviour, in *T. gondii* infection may be explained, in part, by the parasite’s induction of dopaminergic dysregulation. Previous studies have established an association between impulsivity and genetic variations in enzymes related to dopamine transporter activity, dopamine receptors and dopamine metabolic pathways (Ebstein et al. 1996, Retz et al. 2003, Bortolato and Shih 2011, Malloy-Diniz et al. 2013, Chester et al. 2016).

The genome of *T. gondii* contains two genes that encode for tyrosine hydroxylase, the rate-limiting enzyme involved in the production of dopamine (Gaskell et al. 2009). Prior studies have reported heightened concentrations of homovanillic acid, a metabolite of dopamine, as well as enhanced dopamine synthesis in dopaminergic neurons that have been infected with *T. gondii* (Stibbs 1985, Prandovszky et al. 2011, Martin et al. 2015). MicroRNA–132, linked with downregulation of the metabolising enzyme monoamine oxidase A and a decreased expression of D1-like dopamine receptors involved in the negative feedback regulation of dopamine release in the brain (Saklayen et al. 2004), is substantially upregulated by *T. gondii* (Xiao et al. 2014).

The association between a higher magnitude of acoustic startle response and increased dopamine production has also been documented in previous studies (Swerdlow et al. 1986, Parlog et al. 2015), and an increased startle response observed in individuals infected with *T. gondii* has been attributed to the heightened dopamine production in *T. gondii*-positive individuals (Massa et al. 2017). The *T. gondii*−dopamine behavioural connection is further supported by the prevention of *T. gondii*-induced behavioural alterations in animals with anti-dopaminergic agents (Skallová et al. 2006, Webster et al. 2006). In addition to increased production of dopamine, chronic *T. gondii* infection may also inhibit noradrenergic signaling, which could lead to changes in brain connectivity, mood dysregulation, and excessive daytime sleepiness, and potentially contribute to increased risk for suicidal behaviour (Keilp et al. 2013, Lee et al. 2016, Laing et al. 2020).

Results of this study could also be explained by an increased diversion of tryptophan towards the kynurenine pathway rather than the serotonin pathway, as a result of *T. gondii* infection. This could be a consequence of the upregulated immune response attempting to contain the parasite via upregulation of indoleamine 2,3-dioxygenase (IDO), increasing the catabolism of tryptophan via the kynurenine pathway, thus depriving the microorganism of tryptophan, an essential amino acid required for the proliferation of *T. gondii* (Notarangelo et al. 2014).

However, the IDO upregulation results in shunting away of tryptophan from the serotonin pathway with potential for exacerbating depression, impulsivity, and anxiety (Owens and Nemeroff 1994), and from melatonin synthesis with potential sleep-wake dysregulation (Monti 2011). Furthermore, higher plasma levels of kynurenine (Sublette et al. 2011) and cerebrospinal fluid levels of quinolinic acid (an excitotoxic metabolite of the kynurenine pathway) (Erhardt et al. 2013, Brundin et al. 2016) have been reported to be associated with a history of suicide attempt. The kynurenine pathway metabolites have also been directly implicated in depression (Cho et al. 2017) as well as sleep dysregulation (Javelle et al. 2021), both considerable risk factors for suicide.

### Limitations

Major limitations of the current study are the cross-sectional design and lower than expected seropositivity. We decided to only study patients enrolled in mental health treatment for homogeneity and focusing on Veterans most at risk for suicidal behaviour, and who might benefit the most from interventions to decrease their risk. We did not measure *T. gondii* IgM seropositivity and serointensity, even if reported in previous studies in relationship to suicide attempts (Dickerson et al. 2017, Coryell et al. 2020), because the association with IgM (in contrast to IgG) was not confirmed meta-analytically (Amouei et al. 2020).

Additionally, in our previous research (Arling et al. 2009, Okusaga et al. 2011b, Zhang et al. 2012, Cook et al. 2015), we did not confirm associations between *T. gondii* IgM and SSDV or suicide risk factors. Similarly, we have not measured IgG antibodies towards other chronic pathogens, such as CMV (previously reported as associated with psychiatric illness by Burgdorf et al. 2019 and suicidal behaviour by Dickerson et al. 2017, Coryell et al. 2020), as in our hands, we consistently did not find such associations to be significant (Arling et al. 2009, Okusaga et al. 2011b, Zhang et al. 2012, Cook et al. 2015).

Additionally, we did not measure potential autoantibodies that could cross-react with *T. gondii* based on molecular mimicry. Medications with anti-inflammatory properties used by many Veterans (e.g., NSAIDS, certain psychotropic agents) may have blunted certain effects of immune activation in latent toxoplasmosis but may also indicate potential immune mechanisms that may result in cross-reactivity with *T. gondii*. This is potentially most relevant for aspirin given the significantly higher reported use of aspirin in individuals within TQ-Tg-IgG. Other potential risk factors, such as exposure to military and childhood trauma and cognitive dysfunction, represent the focus of two other manuscripts in preparation that will also include biomarkers of inflammation and kynurenine metabolism.

Finally, the secondary hypothesis-driven findings could be an artifact of multiple comparisons. Moreover, these findings may be attributed to reverse causality (i.e., suicide risk factors also elevating risk of *T. gondii* transmission). Future replication is necessary with longitudinal (e.g., with seroconversion), preventative and interventional studies with pharmacological agents that target latent toxoplasmosis are needed from a scientific and clinical perspective.

### Implications

Both pharmacological and psychotherapeutic approaches known to reduce depression, impulsivity, and daytime sleepiness may reduce SSDV-relevant predictive associations of *T. gondii* in US Veterans with a history of a suicide attempt. Furthermore, research into potential anti-*T. gondii* vaccines in humans and primary hosts, and further research into the use of anti-*T. gondii* drugs in individuals who exhibit frequent parasite reactivation with overlapping exacerbation of depression, impulsivity and daytime dysfunction due to sleepiness, with suicidal ideation, may have convergent implications for individualised suicide prevention and risk management.

## Figures and Tables

**Table 1.** Abbreviations Table

Abbreviations	Full Name
AIDS	Acquired Immune Deficiency Syndrome
ANCOVA	Analysis of Covariance
Anti-NMDA	Anti-N-methyl-D-aspartate
BDI-II	Beck Depression Inventory-II
BIS-11	Barratt Impulsiveness Scale Version 11
BPAQ	Buss-Perry Aggression Questionnaire
BSS	Beck Scale for Suicidal Ideation
DSM-5	Diagnostic and Statistical Manual of Mental Disorders, Fifth Edition
EMR	Electronic Medical Records
HIPAA	Health Insurance Portability and Accountability Act
HIV	Human Immunodeficiency Virus
ICD-10	International Classification of Diseases, Tenth Revision
IgG	Immunoglobulin G
IgM	Immunoglobulin M
IRB	Institutional Review Board
L-SASI	Lifetime Suicide Attempt Self Injury Interview
MMSE	Mini-Mental Status Examination
N/A	Not Analysable
NSAID	Non-Steroidal Anti-Inflammatory Drugs
OSU TBI-ID	The Ohio State University Traumatic Brain Injury (TBI) Identification Method
PCL-5	Posttraumatic Stress Disorder Checklist-5
PSQI	Pittsburgh Sleep Quality Index
PTSD	Posttraumatic Stress Disorder
SCID-5-RV	Structured Clinical Interview for Diagnostic and Statistical Manual of Mental Disorders, Fifth Edition, Research Edition
SDV	Self-Directed Violence
SLUMS	Saint Louis University Mental Status
SNRI	Serotonin-Norepinephrine Reuptake Inhibitors
SSDV	Suicidal Self-Directed Violence
SSI	Scale for Suicide Ideation
SSRI	Selective Serotonin Reuptake Inhibitors
TBI	Traumatic Brain Injury
TOMM	Test of Memory Malingering
TQ-Tg-IgG	*Toxoplasma gondii* Top Quartile of Serointensity
UWRAP	University of Washington Risk Assessment Protocol Revised
VA	Veterans Affairs
VHA	Veterans’ Health Administration

**Table 2.** Demographic and socioeconomic variables by SSDV/SDV status and *Toxoplasma gondii* IgG seropositivity status.

Variable	Total	SSDV Positive	SDV Negative	*p*-value	Toxo Positive	Toxo Negative	*p*-value
*N*	407	203	204		25	382	
Age mean (*S.D.*)	45.62 (11.63)	45.55 (11.05)	45.69 (12.21)	*p* = 0.90	49.48 (12.88)	45.37 (11.52)	*p* = 0.09

**Gender**							
Men *N* (%)	304 (74.69)	143 (70.44)	161 (78.92)	*p* = 0.07*	21 (84.00)	283 (74.08)	*p* = 0.29
Women *N* (%)	101 (24.82)	58 (28.57)	43 (21.08)		4 (16.00)	97 (25.39)	
Transgender *N* (%)	2 (0.49)	2 (0.99)	0 (0.00)		0 (0.00)	2 (0.52)	

**Site**							
Atlanta *N* (%)	105 (25.80)	58 (28.57)	47 (23.04)	*p* = 0.22	10 (40.00)	95 (24.87)	*p* = 0.25
Baltimore *N* (%)	118 (28.99)	60 (29.56)	58 (28.43)		6 (24.00)	112 (29.32)	
Denver *N* (%)	184 (45.21)	85 (41.87)	99 (48.53)		9 (36.00)	175 (45.81)	

**Race** ^~^							
Caucasian/White *N* (%)	190 (46.80)	95 (46.80)	95 (46.80)		10 (40.00)	180 (47.24)	
Black/African American *N* (%)	174 (42.86)	90 (44.34)	84 (41.38)		10 (40.00)	164 (43.05)	
Native American/Alaskan Native *N* (%)	5 (1.23)	3 (1.48)	2 (0.99)		0 (0.00)	5 (1.31)	
Asian *N* (%)	1 (0.25)	1 (0.49)	0 (0.00)		0 (0.00)	1 (0.26)	
Pacific Islander *N* (%)	0 (0.00)	0 (0.00)	0 (0.00)		0 (0.00)	0 (0.00)	
Multiracial *N* (%)	28 (6.90)	11 (5.42)	17 (8.37)		3 (12.00)	25 (6.56)	
Other *N* (%)	8 (1.97)	3 (1.48)	5 (2.46)		2(8.00)	6 (1.58)	

**Caucasian**							
Yes *N* (%)	190 (46.68)	95 (46.80)	95 (46.57)	*p* = 0.96	10 (40.00)	180 (47.12)	*p* = 0.49
No *N* (%)	217 (53.32)	108 (53.20)	109 (53.43)		15 (60.00)	202 (52.88)	

**Ethnicity**							
Non-Hispanic/Non-Latino *N* (%)	367 (90.39)	181 (89.16)	186 (91.63)	*p* = 0.40	23 (92.00)	344 (90.29)	*p* = 0.78
Hispanic/Latino *N* (%)	39 (9.61)	22 (10.84)	17 (8.37)		2 (8.00)	37 (9.71)	*p*^#^ = 1.00

**College Graduate**							
Yes *N* (%)	203 (49.88)	92 (45.32)	111 (54.41)	*p* = 0.07*	10 (40.00)	193 (50.52)	*p* = 0.31
No *N* (%)	204 (50.12)	111 (54.68)	93 (45.59)		15 (60.00)	189 (49.48)	

**Marital Status** ^~^							
Married *N* (%)	115 (28.26)	55 (27.09)	60(29.41)		4 (16.00)	111 (29.06)	
Single *N* (%)	140 (34.40)	65 (32.02)	75 (36.77)		15 (60.00)	125 (32.72)	
Cohabitating *N* (%)	31 (7.62)	17 (8.37)	14 (6.86)		3 (12.00)	28 (7.33)	
Widowed *N* (%)	8 (1.97)	4 (1.97)	4 (1.96)		0 (0.00)	8 (2.09)	
Divorced/Separated *N* (%)	113 (27.76)	62 (30.54)	51 (25.00)		3 (12.00)	110 (28.80)	

**Married or Cohabitating**							
Yes *N* (%)	146 (35.87)	72 (35.47)	74 (36.28)	*p* = 0.87	7 (28.00)	139 (36.39)	*p* = 0.40
No *N* (%)	261 (64.13)	131 (64.53)	130 (63.73)		18 (72.00)	243 (63.61)	

**Sexual Orientation** ^~^							
Gay/Lesbian/Queer *N* (%)	20 (4.91)	11 (5.42)	9 (4.41)		1 (4.00)	19 (4.97)	
Heterosexual *N* (%)	375 (92.14)	183 (90.15)	192 (94.12)		24 (96.00)	351 (91.89)	
Bisexual *N* (%)	10 (2.46)	7 (3.45)	3 (1.47)		0 (0.00)	10 (2.62)	
Questioning *N* (%)	0 (0.00)	0 (0.00)	0 (0.00)		0 (0.00)	0 (0.00)	
Other *N* (%)	2 (0.49)	2 (0.985)	0 (0)		0 (0.00)	2 (0.52)	

**Heterosexual**							
Yes *N* (%)	375 (92.14)	183 (90.148)	192 (94.12)	*p* = 0.14	24 (96.00)	351 (91.89)	*p* = 0.46
No *N* (%)	32 (7.86)	20 (9.85)	12 (5.88)		1 (4.00)	31 (8.12)	*p*^#^ = 0.71

**Employment** ^~^							
Employed Full-Time *N* (%)	125 (30.71)	54 (26.60)	71 (34.80)		9 (36.00)	116 (30.37)	
Employed Part-Time *N* (%)	45 (11.06)	21 (10.35)	24 (11.77)		0 (0.00)	45 (11.78)	
Unemployed, not currently seeking employment *N* (%)	103 (25.31)	61 (30.05)	42 (20.59)		9 (36.00)	94 (24.61)	
Unemployed, seeking employment *N* (%)	69 (16.95)	37 (18.22)	32 (15.69)		3 (12.00)	66 (17.28)	
Retired *N* (%)	65 (15.97)	30 (14.78)	35(1716)		4 (16.00)	61 (15.97)	

**Employment or Retired**							
Yes *N* (%)	170 (41.77)	75 (36.95)	95 (46.57)	p = 0.049**	9 (36.00)	161 (42.15)	*p* = 0.55
No *N* (%)	237 (58.23)	128 (63.05)	109 (53.43)		16 (64.00)	221 (57.85)	

**Income**							
< 35,000 *N* (%)	235 (57.74)	71 (34.98)	101 (49.51)	p = 0.003***	21 (84.00)	214 (56.02)	*p* = 0.006**
≥ 35,000 *N* (%)	172 (42.26)	132 (65.03)	103 (50.49)		4 (16.00)	168 (43.98)	*p*^#^ = 0.006**

**Current Student**							
Yes *N* (%)	70 (17.20)	31 (15.27)	39 (19.12)	*p* = 0.30	3 (12.00)	67 (17.54)	*p* = 0.48
No *N* (%)	337 (82.80)	172 (84.73)	165 (80.88)		22 (88.00)	315 (82.46)	*p*^#^ = 0.59

**Current Homelessness**							
Yes *N* (%)	32 (7.86)	24 (11.82)	8 (3.92)	*p* = 0.003***	21 (84.00)	354 (92.67)	*p* = 0.12
No *N* (%)	375 (92.14)	179 (88.19)	196 (96.08)		4 (16.00)	28 (7.33)	*p*^#^ = 0.12

***Toxoplasma* Positive**							
Yes *N* (%)	25 (6.14)	12 (5.91)	13 (6.37)	OR 0.933, *p* = 0.866	25 (100.00)	0 (0.00)’	N/A
No *N* (%)	382 (93.86)	191 (94.09)	191 (93.63)		0 (0.00)	382 (100.00)	

* indicates statistical trend (0.05 < *p* ≤ 0.1);

** indicates statistical significance (0.01 ≤ *p* ≤ 0.05);

*** indicates statistical significance (0.001 < *p* < 0.01); N/A – Not Analysable; SSDV Positive – history of suicidal self-directed violence; SDV Negative – no history of self-directed violence;

~ indicates a variable used for description;

# *p* – indicates Fisher’s exact test *p*-value.

**Table 3.** Military service history by SSDV/SDV status.

Variable	Total	SSDV Positive	SDV Negative	*p*-value	Toxo Positive	Toxo Negative	*p*-value
*N*	407	203	204	N/A	25	382	N/A

**Military History**							
Army Active Duty *N* (%)	206 (50.61)	106 (52.22)	100 (49.02)	*p* = 0.52	18 (72.00)	188 (49.22)	*p* = 0.027**
	
Army Reserves *N* (%)	67 (16.46)	21 (10.35)	46 (22.55)	*p* = 0.001****	8 (32.00)	59 (15.45)	*p* = 0.031**, *p*^#^ = 0.046**
	
Army National Guard *N* (%)	39 (9.58)	22 (10.84)	17 (8.33)	*p* = 0.39	2 (8.00)	37 (9.69)	*p* = 0.781, *p*^#^ = 1.000
	
Air Force Active Duty *N* (%)	52 (12.78)	26 (12.81)	26 (12.75)	*p* = 0.99	2 (8.00)	50 (13.09)	*p* = 0.460, *p*^#^ = 0.756
	
Air Force Reserves *N* (%)	14 (3.44)	8 (3.94)	6 (2.94)	*p* = 0.58	0 (0.00)	14 (3.67)	*p* = 0.330, *p*^#^ = 1.000
	
Air Force National Guard *N* (%)	8 (1.97)	2 (0.99)	6 (2.94)	*p* = 0.16	1 (4.00)	7(1.83)	*p* = 0.449, *p*^#^ = 0.401
	
Navy Active Duty *N* (%)	73 (17.94)	38 (18.72)	35 (17.16)	*p* = 0.68	2 (8.00)	71 (18.59)	*p* = 0.181, *p*^#^ = 0.280
	
Navy Reserves *N* (%)	15 (3.69)	6 (2.96)	9 (4.41)	*p* = 0.44	1 (4.00)	14 (3.67)	*p* = 0.931, *p*^#^ = 1.000
	
Marine Active Duty *N* (%)	62 (15.23)	27 (13.3)	35 (17.16)	*p* = 0.28	3 (12.00)	59 (15.45)	*p* = 0.642, *p*^#^ = 0.781
	
Marine Reserves *N* (%)	10 (2.46)	1 (0.49)	9 (4.41)	*p* = 0.020**	0 (0.00)	10 (2.62)	*p* = 0.413, *p*^#^ = 1.000
	
Coast Guard Active Duty *N* (%)	1 (0.25)	1 (0.49)	0 (0.00)	*p* = 0.32	0 (0.00)	1 (0.26)	*p* = 0.798, *p*^#^ = 1.000
	
Coast Guard Reserves *N* (%)	0 (0.00)	0 (0.00)	0 (0.00)		0 (0.00)	0 (0.00)	
	
Service Era: Vietnam *N* (%)	13 (3.19)	5 (2.46)	8 (3.92)	*p* = 0.40	0 (0.00)	13 (3.40)	*p* = 0.349, *p*^#^ = 1.000
	
Service Era: Post-Vietnam *N* (%)	154 (37.84)	79 (38.92)	75 (36.77)	*p* = 0.66	14 (56.00)	140 (36.65)	*p* = 0.053*, *p*^#^ = 0.058*
	
Service Era: Desert-Storm *N* (%)	139 (34.15)	69 (34.15)	70 (34.31)	*p* = 0.95	4 (16.00)	135 (35.34)	*p* = 0.048**, *p* = 0.052*
	
Service Era: OEF/OIF/OND *N* (%)	211 (51.84)	99 (48.77)	112 (54.90)	*p* = 0.22	10 (40.00)	201 (52.62)	*p* = 0.221, *p*^#^ = 0.302
	
Rank: Enlisted *N* (%)	260 (63.88)	141 (69.46)	119 (58.33)	*p* = 0.06*	19 (76.00)	241 (63.09)	*p* = 0.331
		
Rank: Non-Commissioned Officer *N* (%)	131 (32.19)	56 (27.59)	75 (36.77)		6 (24.00)	125 (32.72)	
		
Rank: Officer *N* (%)	16 (3.93)	6 (2.96)	10 (4.90)		0 (0.00)	16 (4.19)	

**Officer**							
Yes *N* (%)	147 (36.12)	62 (30.54)	82 (41.67)	*p* = 0.019**	6 (24.00)	141 (36.91)	*p* = 0.193, *p*^#^ = 0.282
		
No *N* (%)	391 (63.88)	197 (69.46)	194 (58.33)		19 (76.00)	241 (63.09)	
	
Received uninvited/unwanted sexual attention *N* (%)	139 (34.15)	86 (42.37)	53 (25.98)	*p* = 0.097*	3 (12.00)	136 (35.60)	*p* = 0.016**, *p*^#^ = 0.016**
	
Did anyone ever use force/the threat of force to have sexual contact with you against your will *N* (%)	84 (20.64)	54 (26.60)	30 (14.71)	*p* = <0.001****	1 (4.00)	83 (21.73)	*p* = 0.034**, *p*^#^ = 0.038**
	
Months of Active-Duty Service (*S.D.*)	74.39 ± 58.93	68.46 ± 54.69	80.26 ± 62.44	*p* = 0.043**	64.24 ± 36.31	75.05 ± 60.09	*p* = 0.375
	
Months of Reserve Service (*S.D.*)	23.10 ± 53.52	14.11 ± 37.12	32.09 ± 64.83	*p* = 0.001****	20.960 ± 34.52	23.24 ± 54.57	*p* = 0.837
	
# of deployments (*S.D.*)	1.77 ± 3.21	1.44 ± 1.83	2.09 ± 4.13	*p* = 0.039**	1.16 ± 1.11	1.81 ± 3.30	*p* = 0.330
	
# of combat tours (*S.D.*)	0.80 ± 1.25	0.65 ± 1.00	0.95 ± 1.44	*p* = 0.015**	0.44 ± 0.65	0.82 ± 1.27	*p* = 0.141

* indicates statistical trend (0.05 < *p* ≤ 0.1);

** indicates statistical significance (0.01 ≤ *p* ≤ 0.05);

*** indicates statistical significance (0.001 < *p* < 0.01);

**** indicates statistical significance (*p* ≤ 0.001); N/A – Not Analysable; SSDV Positive – history of suicidal self-directed violence; SDV Negative – no history of self-directed violence;

# *p* – indicates Fisher’s exact test was used.

**Table 4.** Mental health and substance use disorders by SSDV/SDV status and *Toxoplasma gondii* IgG seropositivity

Variable	Total	SSDV Positive	SDV Negative	*p*-value	Toxo Positive	Toxo Negative	*p*-value
*N*	407	203	204	N/A	25	382	N/A
	
Age (in years): Mean (*S.D.*)	45.62 (11.63)	45.55 (11.05)	45.69 (12.21)	N/A	49.48 (12.86)	45.37 (11.52)	*p* = 0.087*
	
Any Bipolar Disorder in Lifetime *N* (%)	50 (12.29)	36 (17.73)	14 (6.86)	*p* = 0.001****	1 (4.00)	49 (12.83)	*p* = 0.193, *p*^#^ = 0.341
	
Any Depressive Disorder in Lifetime *N* (%)	291 (71.50)	155 (76.36)	136 (66.67)	*p* = 0.030**	17 (68.00)	274 (71.73)	*p* = 0.689
	
Any Major Depressive Disorder in Lifetime *N* (%)	269 (66.09)	145 (71.43)	124 (60.78)	*p* = 0.023**	14 (56.00)	255 (66.75)	*p* = 0.271
	
Any Schizophrenia in Lifetime *N* (%)	15 (3.69)	9 (4.43)	6 (2.94)	*p* = 0.424^#^	2 (8.00)	13 (3.40)	*p* = 0.237, *p*^#^ = 0.233
	
Any Substance Use Disorder in Lifetime *N* (%)	315 (77.40)	167 (82.27)	148 (72.55)	*p* = 0.019**	21 (84.00)	294 (76.96)	*p* = 0.415
	
Any Alcohol Disorder prior to past 12 months *N* (%)	296 (72.73)	158 (77.83)	138 (67.65)	*p* = 0.021**	19 (76.00)	277 (72.51)	*p* = 0.704
	
Any Sedative-Hypnotic Anxiolytic Use prior to past 12 months *N* (%)	21 (5.17)	15 (7.43)	6 (2.94)	*p* = 0.041**	1 (4.00)	20 (5.25)	*p* = 0.785, *p*^#^ = 1.000
	
Any Cannabis Use prior to past 12 months *N* (%)	140 (34.57)	84 (41.58)	56 (27.57)	*p* = 0.003***	13 (52.00)	127 (33.42)	*p* = 0.058*
	
Any Stimulant Use prior to past 12 months *N* (%)	93 (22.91)	58 (28.71)	35 (17.16)	*p* = 0.006***	7 (28.00)	86 (22.57)	*p* = 0.532
	
Any Opioid Use prior to past 12 months *N* (%)	33 (8.11)	22 (10.84)	11 (5.39)	*p* = 0.044**	1 (4.00)	32 (8.38)	*p* = 0.437, *p*^#^ = 0.709
	
Any Hallucinogens prior to past 12 months^¥^ *N* (%)	16 (3.94)	11 (5.45)	5 (2.45)	*p* = 0.121^#^	0 (0.00)	16 (4.20)	*p* = 0.296, *p*^#^ = 0.612
	
Any Panic Disorder in Lifetime *N* (%)	64 (15.84)	41 (20.30)	23 (11.39)	*p* = 0.014**	1 (4.00)	63 (16.62)	*p* = 0.094, *p*^#^ = 0.152
	
Any Agoraphobia in Lifetime *N* (%)	17 (4.19)	15 (7.39)	2 (0.99)	*p* = 0.001^#^****	1 (4.00)	16 (4.20)	*p* = 0.962, *p*^#^ = 1.000
	
Any Social Anxiety Disorder in Lifetime *N* (%)	31 (7.62)	24 (11.82)	7 (3.43)	*p* = 0.001****	2 (8.00)	29 (7.60)	*p* = 0.941, *p*^#^ = 1.000
	
Any Generalized Anxiety Disorder in Lifetime *N* (%)	62 (15.27)	39 (19.31)	23 (11.28)	*p* = 0.024**	2 (8.00)	60 (15.75)	*p* = 0.297, *p*^#^ = 0.398
	
Any Intermittent Explosive Disorder in past 12 months *N* (%)	20 (4.99)	14 (7.00)	6 (2.99)	*p* = 0.065^#^*	2 (8.00)	18 (4.79)	*p* = 0.475, *p*^#^ = 0.359
	
Any Posttraumatic Stress Disorder in Lifetime *N* (%)	219 (54.21)	124 (62.00)	95 (46.57)	*p* = 0.002***	8 (32.00)	211 (55.67)	*p* = 0.021**, *p*^#^ = 0.024**
	
Any Posttraumatic Stress Disorder in past month *N* (%)	148 (36.63)	89 (44.50)	59 (28.92)	*p* = 0.001****	7 (28.00)	141 (37.20)	*p* = 0.355, *p*^#^ = 0.399
	
Any Mild Traumatic Brain Injury *N* (%)	249 (61.18)	138 (67.98)	111 (54.41)	*p* = 0.005***	13 (52.00)	236 (61.78)	*p* = 0.331

# Fisher’s Exact Test was used to find *p*-value for *n* = 20 variables and below;

¥ indicates other hallucinogens apart from phencyclidine (PCP);

* indicates statistical trend (0.05 < *p* ≤ 0.1);

** indicates statistical significance (0.01 ≤ *p* ≤ 0.05);

*** indicates statistical significance (0.001 < *p* < 0.01);

**** indicates statistical significance (*p* ≤ 0.001); N/A – Not Analysable; SSDV Positive – history of suicidal self-directed violence; SDV Negative – no history of self-directed violence.

**Table 5.** Medications by history of self-directed violence and *Toxoplasma gondii* IgG seropositivity

Variable	Total	SSDV Positive	SDV Negative	*p*-value	Toxo Positive	Toxo Negative	*p*-value
*N*	407	203	204	N/A	25	382	N/A
	
*N* (%) with valid medication data	385 (94.59)	192 (94.58)	193 (94.61)	N/A	24 (96.00)	361 (94.50)	N/A
	
Age (in years): Mean (*S.D.*)	45.62 (11.63)	45.55 (11.05)	45.69 (12.21)	N/A	49.48 (12.86)	45.37 (11.52)	*p* = 0.087*
	
Any Psychotropic Medication *N* (%)	185 (48.05)	101 (52.60)	84 (43.52)	*p* = 0.075*	14 (58.33)	171 (47.37)	*p* = 0.298
	
Any Anticholinergic Medication *N* (%)	17 (4.43)	12 (6.28)	5 (2.59)	*p* = 0.088^#^*	1 (4.17)	16 (4.44)	*p* = 0.949, *p*^#^ = 1.000
	
Any Second-generation Antipsychotic *N* (%)	44 (11.43)	31 (16.15)	13 (6.74)	*p* = 0.004***	3 (12.50)	41 (11.36)	*p* = 0.865, *p*^#^ = 0.746
	
Any First-generation Antipsychotic *N* (%)	6 (1.56)	3 (1.56)	3 (1.55)	*p* = 1.000^#^	1 (4.17)	5 (1.39)	*p* = 0.287, *p*^#^ = 0.322
	
Clozapine *N* (%)	1 (0.26)	1 (0.52)	0 (0.00)	N/A	0 (0.00)	1 (0.28)	N/A
	
Any Benzodiazepine *N* (%)	13 (3.38)	8 (4.17)	5 (2.59)	*p* = 0.415^#^	1 (4.17)	12 (3.32)	*p* = 0.825, *p*^#^ = 0.573
	
Any Other Anxiolytics *N* (%)	49 (12.73)	27 (14.06)	22 (11.40)	*p* = 0.433	1 (4.17)	48 (13.30)	*p* = 0.194, *p*^#^ = 0.339
	
Lithium *N* (%)	15 (3.90)	12 (6.25)	3 (1.55)	*p* = 0.019^#^**	0 (0.00)	15 (4.16)	*p* = 0.308, *p*^#^ = 0.612
	
Any Other Mood Stabilizers *N* (%)	19 (4.94)	14 (7.29)	5 (2.59)	*p* = 0.036^#^**	2 (8.33)	17 (4.71)	*p* = 0.427, *p*^#^ = 0.335
	
Prazosin *N* (%)	18 (4.68)	9 (4.69)	9 (4.66)	*p* = 1.000^#^	0 (0.00)	18 (4.99)	*p* = 0.263, *p*^#^ = 0.616
	
Any SSRI *N* (%)	77 (20.00)	45 (23.44)	32 (16.58)	*p* = 0.093*	5 (20.83)	72 (19.95)	*p* = 0.916, *p*^#^ = 1.000
	
Any SNRI *N* (%)	35 (9.09)	21 (10.94)	14 (7.25)	*p* = 0.209	4 (16.67)	31 (8.59)	*p* = 0.182, *p*^#^ = 0.257
	
Mirtazapine *N* (%)	15 (3.90)	10 (5.21)	5 (2.59)	*p* = 0.200^#^	2 (8.33)	13 (3.60)	*p* = 0.246, *p*^#^ = 0.238
	
Bupropion *N* (%)	28 (7.27)	17 (8.85)	11 (5.70)	*p* = 0.233	1 (4.17)	27 (7.48)	*p* = 0.545, *p*^#^ = 1.000
	
Other Antidepressants *N* (%)	52 (13.51)	31 (16.15)	21 (10.88)	*p* = 0.131	4 (16.67)	48 (13.30)	*p* = 0.640, *p*^#^ = 0.549
	
Trazodone *N* (%)	48 (12.47)	31 (16.15)	17 (8.81)	*p* = 0.029**	3 (12.50)	45 (12.47)	*p* = 0.996, *p*^#^ = 1.000
	
Any Other Insomnia Medications *N* (%)	54 (14.03)	25 (13.02)	29 (15.03)	*p* = 0.571	3 (12.50)	51 (14.13)	*p* = 0.824, *p*^#^ = 1.000
	
Any NSAID *N* (%)	86 (22.34)	39 (20.31)	47 (24.35)	*p* = 0.341	5 (20.83)	81 (22.44)	*p* = 0.855, *p*^#^ = 1.000
	
Aspirin *N* (%)	35 (9.11)	16 (8.38)	19 (9.84)	*p* = 0.617	4 (16.67)	31 (8.61)	*p* = 0.184, *p*^#^ = 0.258
	
Ibuprofen *N* (%)	32 (8.31)	12 (6.25)	20 (10.36)	*p* = 0.144	1 (4.17)	31 (8.59)	*p* = 0.447, *p*^#^ = 0.708
	
Any Other NSAIDs^+^ *N* (%)	22 (5.71)	13 (6.77)	9 (4.66)	*p* = 0.373	0 (0.00)	22 (6.09)	*p* = 0.213, *p*^#^ = 0.382
	
Any Statin *N* (%)	45 (11.69)	17 (8.85)	28 (14.51)	*p* = 0.084*	2 (8.33)	43 (11.91)	*p* = 0.597, *p*^#^ = 1.000
	
Any Hydrophilic Statin *N* (%)	11 (2.86)	5 (2.60)	6 (3.11)	*p* = 1.000^#^	0 (0.00)	11 (3.05)	*p* = 0.386, *p*^#^ = 1.000
	
Any Lipophilic Statin *N* (%)	33 (8.57)	12 (6.25)	21 (10.88)	*p* = 0.105	2 (8.33)	31 (8.59)	*p* = 0.966, *p*^#^ = 1.000

* indicates statistical trend (0.05 < *p* ≤ 0.1);

** indicates statistical significance (0.01 ≤ *p* ≤ 0.05);

*** indicates statistical significance (0.001 < *p* < 0.01);

**** indicates statistical significance (*p* ≤ 0.001);

+ indicates all other NSAIDS besides aspirin and ibuprofen;

# *P*-value was calculated with the Fisher’s Exact Test; the percentages were calculated on 385/407 (94.59%) participants with available specific medication data; IgG – Immunoglobulin G; N/A – Not Analysable; NSAID – Non-Steroidal Anti-Inflammatory Drugs; SSDV Positive – history of suicidal self-directed violence; SDV Negative – no history of self-directed violence; SNRI – Serotonin-Norepinephrine Reuptake Inhibitors; SSRI – Selective Serotonin Reuptake Inhibitors.

**Table 6.** Mental health and substance use disorders in participants within top quartile vs bottom three quartiles of *Toxoplasma gondii* IgG serointensity.

Variable	Total	*T. gondii* IgG Top quartile	*T. gondii* IgG Bottom three quartiles	*p*-value
*N*	407	115	292	N/A
Age (in years): Mean (*S.D.*)	45.62 (11.63)	48.80 (11.28)	44.37 (11.55)	N/A
Any Bipolar Disorder in Lifetime *N* (%)	50 (12.29)	14 (12.17)	36 (12.33)	*p* = 0.966
Any Depressive Disorder in Lifetime *N* (%)	291 (71.50)	86 (74.78)	205 (70.21)	*p* = 0.357
Any Major Depressive Disorder in Lifetime *N* (%)	269 (66.09)	78 (67.83)	191 (65.41)	*p* = 0.643
Any Schizophrenia in Lifetime *N* (%)	15 (3.69)	4 (3.48)	11 (3.77)	*p* = 0.889^#^
Any Substance Use Disorder in Lifetime *N* (%)	315 (77.40)	89 (77.39)	226 (77.40)	*p* = 0.999
Any Alcohol Disorder prior to past 12 months *N* (%)	296 (72.73)	81 (70.44)	215 (73.63)	*p* = 0.515
Any Sedative-Hypnotic Anxiolytic Use prior to past 12 months *N* (%)	21 (5.17)	5 (4.35)	16 (5.50)	*p* = 0.637
Any Cannabis Use prior to past 12 months *N* (%)	140 (34.57)	41 (35.65)	99 (34.14)	*p* = 0.773
Any Stimulant Use prior to past 12 months *N* (%)	93 (22.91)	33 (28.70)	60 (20.62)	*p* = 0.081*
Any Opioid Use prior to past 12 months *N* (%)	33 (8.11)	6 (5.22)	27 (9.25)	*p* = 0.180
Any Hallucinogens prior to past 12 months^¥^ *N* (%)	16 (3.94)	6 (5.22)	10 (3.44)	*p* = 0.406^#^
Any Panic Disorder in Lifetime *N* (%)	64 (15.84)	17 (15.04)	47 (16.15)	*p* = 0.784
Any Agoraphobia in Lifetime *N* (%)	17 (4.19)	3 (2.63)	14 (4.80)	*p* = 0.328^#^
Any Social Anxiety Disorder in Lifetime *N* (%)	31 (7.62)	10 (8.70)	21 (7.19)	*p* = 0.607
Any Generalized Anxiety Disorder in Lifetime *N* (%)	62 (15.27)	19 (16.52)	43 (14.78)	*p* = 0.660
Any Intermittent Explosive Disorder in past 12 months *N* (%)	20 (4.99)	6 (5.36)	14 (4.84)	*p* = 0.832^#^
Any Posttraumatic Stress Disorder in Lifetime *N* (%)	219 (54.21)	57 (50.00)	162 (55.86)	*p* = 0.287
Any Posttraumatic Stress Disorder in past month *N* (%)	148 (36.63)	41 (35.97)	107 (36.90)	*p* = 0.861
Any Mild Traumatic Brain Injury *N* (%)	249 (61.18)	68 (59.13)	181 (61.97)	*p* = 0.595

# Fisher’s Exact Test was used to find *p*-value for *n* = 20 variables and below;

¥ indicates other hallucinogens apart from phencyclidine (PCP);

* indicates statistical trend (0.05 < *p* ≤ 0.1); IgG – Immunoglobulin G; N/A – Not Analysable.

**Table 7.** Medications in participants within the Top quartile vs. Bottom three quartiles of *Toxoplasma gondii* IgG serointensity.

Variable	Total	*T. gondii* IgG Top quartile	*T. gondii* IgG Bottom three quartiles	*p*-value
*N*	407	115	292	N/A
*N* with valid medication data	385	110	275	N/A
Age (in years): Mean (*S.D.*)	45.62 (11.63)	48.80 (11.28)	44.37 (11.55)	N/A
Any Psychotropic Medication *N* (%)	185 (48.05)	62 (56.36)	123 (44.73)	*p* = 0.039**
Any Anticholinergic Medication^¶^ *N* (%)	17 (4.43)	6 (5.46)	11 (4.02)	*p* = 0.585^#^
Any Second-generation Antipsychotic *N* (%)	44 (11.43)	14 (12.73)	30 (10.91)	*p* = 0.612
Any Typical Antipsychotic *N* (%)	6 (1.56)	3 (2.73)	3 (1.09)	*p* = 0.359^#^
Clozapine *N* (%)	1 (0.26)	0 (0.000)	1 (0.36)	N/A
Any Benzodiazepine *N* (%)	13 (3.38)	5 (4.55)	8 (2.91)	*p* = 0.532^#^
Any Other Anxiolytics *N* (%)	49 (12.73)	16 (14.55)	33 (12.00)	*p* = 0.498
Lithium *N* (%)	15 (3.90)	5 (4.55)	10 (3.64)	*p* = 0.771^#^
Any Other Mood Stabilizers *N* (%)	19 (4.94)	12 (10.91)	7 (2.55)	*p* = 0.001^#^****
Prazosin *N* (%)	18 (4.68)	4 (3.64)	14 (5.09)	*p* = 0.790^#^
Any SSRI *N* (%)	77 (20.00)	24 (21.82)	53 (19.27)	*p* = 0.573
Any SNRI *N* (%)	35 (9.09)	13 (11.82)	22 (8.00)	*p* = 0.239
Mirtazapine *N* (%)	15 (3.90)	4 (3.64)	11 (4.00)	*p* = 1.000^#^
Bupropion *N* (%)	28 (7.27)	11 (10.00)	17 (6.18)	*p* = 0.192
Other Antidepressants *N* (%)	52 (13.51)	17 (15.46)	35 (12.73)	*p* = 0.479
Trazodone *N* (%)	48 (12.47)	14 (12.73)	34 (12.36)	*p* = 0.922
Any Other Insomnia Medications *N* (%)	54 (14.03)	19 (17.27)	35 (12.73)	*p* = 0.246
Any NSAID *N* (%)	86 (22.34)	31 (28.18)	55 (20.00)	*p* = 0.082*
Aspirin *N* (%)	35 (9.11)	17 (15.46)	18 (6.57)	*p* = 0.006***
Ibuprofen *N* (%)	32 (8.31)	10 (9.09)	22 (8.00)	*p* = 0.726
Any Other NSAIDs^+^ *N* (%)	22 (5.71)	5 (4.55)	17 (6.18)	*p* = 0.532
Any Statin *N* (%)	45 (11.69)	18 (16.36)	27 (9.82)	*p* = 0.071*
Any Hydrophilic Statin *N* (%)	11 (2.86)	5 (4.55)	6 (2.18)	*p* = 0.307^#^
Any Lipophilic Statin *N* (%)	33 (8.57)	13 (11.82)	20 (7.27)	*p* = 0.150

* indicates statistical trend (0.05 < *p* ≤ 0.1);

** indicates statistical significance (0.01 ≤ *p* ≤ 0.05);

*** indicates statistical significance (0.001 < *p* < 0.01);

**** indicates statistical significance (*p* ≤ 0.001);

+ indicates all other NSAIDS besides aspirin and ibuprofen;

# *P*-value was calculated with the Fisher’s Exact Test; IgG – Immunoglobulin G; N/A – Not Analysable; NSAID – Non-Steroidal Anti-Inflammatory Drugs.

**Table 8.** Associations between *Toxoplasma gondii* IgG seropositivity and suicide risk factors in total, SSDV Positive only, and SDV Negative only samples

Sample Type	Total Sample			SSDV Positive			SDV Negative		
Variable	Unadjusted	Adjusted Age	Adjusted Age+Gender	Unadjusted	Adjusted Age	Adjusted Age+Gender	Unadjusted	Adjusted Age	Adjusted Age+Gender
Attempted Suicide	OR 0.933, 95% CI (0.415, 2.097), *p* = 0.866	OR 0.934, 95% CI (0.414, 2.106), *p* = 0.870	OR 0.975, 95% CI (0.431, 2.206), *p* = 0.952	N/A	N/A	N/A	N/A	N/A	N/A
	
Multiple Suicide Attempts vs Single	N/A	N/A	N/A	OR 1.434, 95% CI (0.417, 4.935), *p* = 0.568	OR 1.352, 95% CI (0.389, 4.698), *p* = 0.635	OR 1.426, 95% CI (0.408, 4.980), *p* = 0.578	N/A	N/A	N/A
	
BSS Score Rank Transformed	*F*_(1,400)_ = 0.003, *p* = 0.956	*F*_(1,399)_ = 0.009, *p* = 0.926	*F*_(1,397)_ = 0.094, *p* = 0.760	*F*_(1,198)_ = 0.401, *p* = 0.528	*F*_(1,197)_ = 0.669, *p* = 0.414	*F*_(1,195)_ = 0.578, *p* = 0.488	*F*_(1,200)_ = 1.521, *p* = 0.219	*F*_(1,199)_ = 1.370, *p* = 0.243	*F*_(1,197)_ = 0.274, *p* = 0.601
	
BIS Total Score Top Quartile	OR 0.979, 95% CI (0.398, 2.412), *p* = 0.964	OR 1.036, 95% CI (0.418, 2.566), *p* = 0.939	1.093, 95% CI (0.439, 2.721), *p* = 0.848	OR 1.110, 95% CI (0.340, 3.628), *p* = 0.863	OR 1.219, 95% CI (0.368, 4.042), *p* = 0.746	OR 1.301, 95% CI (0.389, 4.354), *p* = 0.670	OR 0.840, 95% CI (0.178, 3.962), *p* = 0.825	OR 0.869, 95% CI (0.183, 4.119), *p* = 0.859	OR 0.890, 95% CI (0.187, 4.230), *p* = 0.883
	
BPAQ Physical Aggression Factor Score Total	*F*_(1,403)_ = 2.432, *p* = 0.120	*F*_(1,402)_ = 1.620, *p* = 0.204	*F*_(1,400)_ = 0.323, *p* = 0.570	*F*_(1,199)_ = 0.779, *p* = 0.379	*F*_(1,198)_ = 0.147, *p* = 0.702	*F*_(1,196)_ = 0.167, *p* = 0.683	*F*_(1,202)_ = 1.778, *p* = 0.184	*F*_(1,201)_ = 1.675, *p* = 0.197	*F*_(1,199)_ = 1.564, *p* = 0.213
	
BPAQ Anger Factor Score Total	*F*_(1,403)_ = 2.011, *p* = 0.157	*F*_(1,402)_ = 1.649, *p* = 0.200	*F*_(1,400)_ = 0.713, *p* = 0.399	*F*_(1,199)_ = 0.484, *p* = 0.487	*F*_(1,198)_ = 0.145, *p* = 0.704	*F*_(1,196)_ = 0.071, *p* = 0.790	*F*_(1,202)_ = 1.753, *p* = 0.187	*F*_(1,201)_ = 1.826, *p* = 0.178	*F*_(1,199)_ = 0.924, *p* = 0.338
	
BPAQ Verbal Aggression Factor Score Total	*F*_(1,403)_ = 0.011, *p* = 0.917	*F*_(1,402)_ = 0.006, *p* = 0.937	*F*_(1,400)_ = 0.607, *p* = 0.436	*F*_(1,199)_ = 0.946, *p* = 0.332	*F*_(1,198)_ = 0.403, *p* = 0.527	*F*_(1,196)_ = 1.073, *p* = 0.301	*F*_(1,202)_ = 0.642, *p* = 0.424	*F*_(1,201)_ = 0.652, *p* = 0.421	*F*_(1,199)_ = 0.008, *p* = 0.927
	
BPAQ Hostility Factor Score Total	*F*_(1,403)_ = 1.446, *p* = 0.230	*F*_(1,402)_ = 1.179, *p* = 0.278	*F*_(1,400)_ = 0.157, *p* = 0.693	*F*_(1,199)_ = 0.309, *p* = 0.579	*F*_(1,198)_ = 0.097, *p* = 0.755	*F*_(1,196)_ = 1.198, *p* = 0.275	*F*_(1,202)_ = 1.272, *p* = 0.261	*F*_(1,201)_ = 1.273, *p* = 0.260	*F*_(1,199)_ = 0.185, *p* = 0.667
	
BPAQ Score Total	*F*_(1,403)_ = 2.055, *p* = 0.152	*F*_(1,402)_ = 1.474, *p* = 0.225	*F*_(1,400)_ = 0.245, *p* = 0.621	*F*_(1,199)_ = 0.834, *p* = 0.362	*F*_(1,198)_ = 0.241, *p* = 0.624	*F*_(1,196)_ = 0.045, *p* = 0.833	*F*_(1,202)_ = 1.248, *p* = 0.265	*F*_(1,201)_ = 1.232, *p* = 0.268	*F*_(1,199)_ = 0.841, *p* = 0.360
	
Log of BDI Score Total	*F*_(1,401)_ = 0.037, p = 0.848	*F*_(1,400)_ = 0.009, p = 0.925	*F*_(1,401)_ = 0.050, *p* = 0.823	*F*_(1,198)_ = 0.089, *p* = 0.765	*F*_(1,197)_ = 0.309, *p* = 0.579	*F*_(1,195)_ = 0.495, *p* = 0.483	*F*_(1,201)_ = 0.346, *p* = 0.557	*F*_(1,200)_ = 0.390, *p* = 0.533	*F*_(1,198)_ = 1.303, *p* = 0.255
	
Depressed by BDI Score ≥14	OR 0.649, 95% CI (0.287, 1.467), *p* = 0.299	OR 0.661, 95% CI (0.292, 1.499), *p* = 0.322	OR 0.704, 95% CI (0.308, 1.608), *p* = 0.405	OR 0.723, 95% CI (0.221, 2.367), *p* = 0.591	OR 0.867, 95% CI (0.257, 2.931), *p* = 0.818	OR 0.925, 95% CI (0.271, 3.161), *p* = 0.901	OR 0.573, 95% CI (0.170, 1.926), *p* = 0.368	OR 0.539, 95% CI (0.159, 1.831), *p* = 0.322	OR 0.560, 95% CI (0.162, 1.932), *p* = 0.359
	
Sleep Disturbance	OR 0.744, 95% CI (0.325, 1.704), *p* = 0.484	OR 0.692, 95% CI (0.300, 1.597), *p* = 0.388	OR 0.720, 95% CI (0.311, 1.667), *p* = 0.443	OR 0.345, 95% CI (0.106, 1.122), *p* = 0.077*↓	OR 0.323, 95% CI (0.098, 1.064), *p* = 0.063* ↓	OR 0.335, 95% CI (0.101, 1.110), *p* = 0.074* ↓	OR 1.533, 95% CI (0.456, 5.156), *p* = 0.490	OR 1.449, 95% CI (0.426, 4.933), *p* = 0.553	OR 1.493, 95% CI (0.436, 5.110), *p* = 0.523
	
Prolonged Sleep Latency	OR 0.792, 95% CI (0.308, 2.038), *p* = 0.629	OR 0.845, 95% CI (0.326, 2.187), *p* = 0.728	OR 0.856, 95% CI (0.330, 2.218), *p* = 0.748	OR 1.429, 95% CI (0.436, 4.681), *p* = 0.556	OR 1.689, 95% CI (0.501, 5.698), *p* = 0.398	OR 1.710, 95% CI (0.505, 5.791), *p* = 0.389	OR 0.269, 95% CI (0.034, 2.122), *p* = 0.213	OR 0.272, 95% CI (0.034, 2.153), *p* = 0.217	OR 0.273, 95% CI (0.034, 2.159), *p* = 0.218
	
Daytime Dysfunction due to Sleepiness	OR 1.060, 95% CI (0.386, 2.914), *p* = 0.909	1.182, 95% CI (0.425, 3.288), *p* = 0.748	1.232, 95% CI (0.442, 3.454), *p* = 0.690	OR 0.949, 95% CI (0.198, 4.552), *p* = 0.948	OR 1.351, 95% CI (0.266, 6.853), *p* = 0.717	1.402, 95% CI (0.274, 7.177), *p* = 0.685	OR 1.167, 95% CI (0.308, 4.414), *p* = 0.820	OR 1.819, 95% CI (0.313, 4.514), *p* = 0.799	OR 1.223, 95% CI (0.321, 4.655), *p* = 0.768
	
Impaired Sleep Efficiency	OR 0.554, 95% CI (0.216, 1.420), *p* = 0.219	OR 0.581, 95% (0.226, 1.495), *p* = 0.260	OR 0.574, 95% CI (0.223, 1.479), *p* = 0.250	OR 0.261, 95% CI (0.056, 1.224), *p* = 0.088* ↓	OR 0.283, 95% CI (0.060, 1.336), *p* = 0.111	OR 0.285, 95% CI (0.060, 1.348), *p* = 0.113	OR 1.071, 95% CI (0.317, 3.623), *p* = 0.912	OR 1.097, 95% CI (0.323, 3.724), *p* = 0.882	OR 1.059, 95% CI (0.310, 3.614), *p* = 0.927
	
Impaired Overall Sleep Quality	OR 0.632, 95% CI (0.277, 1.443), *p* = 0.276	OR 0.683, 95% CI (0.297, 1.572), *p* = 0.370	OR 0.680, 95% CI (0.295, 1.567), *p* = 0.365	OR 0.255, 95% CI (0.067, 0.974), *p* = 0.046** ↓	OR 0.283, 95% CI (0.073, 1.092), *p* = 0.067* ↓	OR 0.290, 95% CI (0.075, 1.123), *p* = 0.073* ↓	OR 1.366, 95% CI (0.442, 4.214), *p* = 0.588	OR 1.446, 95% CI (0.464, 4.508), *p* = 0.525	OR 1.406, 95% CI (0.448, 4.414), *p* = 0.559
	
PSQI Total Score	*F*_(1,397)_ = 0.266, *p* = 0.606	*F*_(1,396)_ = 0.183, *p* = 0.669	*F*_(1,394)_ = 0.000, *p* = 0.993	*F*_(1,195)_ = 1.736, *p* = 0.189	*F*_(1,194)_ = 1.185, *p* = 0.278	*F*_(1,192)_ = 0.072, *p* = 0.789	*F*_(1,200)_ = 0.318, *p* = 0.573	*F*_(1,199)_ = 0.288, *p* = 0.592	*F*_(1,197)_ = 0.052, *p* = 0.821

* indicates statistical trend (0.05 < *p* ≤ 0.1);

** indicates statistical significance (0.01 ≤ *p* ≤ 0.05);

↑ indicates a positive association between the variable and *T. gondii* seropositivity (higher in *T. gondii* seropositive cases);

↓ indicates a negative association between the variable and *T. gondii* seropositivity (lower in the *T. gondii* seropositive cases); OR > 1 indicates a positive association (variable is higher in *T. gondii* seropositive cases); OR < 1 indicates a negative association (variable is lower in *T. gondii* seropositive cases);

* indicates statistical trend (0.05 < *p* ≤ 0.1); BDI – Beck Depression Inventory; BIS – Barratt Impulsiveness Scale Version 11; BPAQ – Buss-Perry Aggression Questionnaire; BSS – Beck Scale for Suicidal Ideation; N/A – Not Analysable; PSQI – Pittsburgh Sleep Quality Index

**Table 9.** Analysis of the association between *Toxoplasma gondii* IgG high versus low serointensity groups (high quartile versus bottom three quartiles) and Suicide Risk Factors in total, SSDV Positive, and SDV Negative samples

Sample Type	Total Sample			SSDV Positive			SDV Negative		
Variable	Unadjusted	Adjusted Age	Adjusted Age+Gender	Unadjusted	Adjusted Age	Adjusted Age+Gender	Unadjusted	Adjusted Age	Adjusted Age+Gender
Attempted Suicide	OR 1.126, 95% CI (0.730, 1.736), *p* = 0.593	OR 1.133, 95% CI (0.729, 1.759), *p* = 0.579)	OR 1.105, 95% CI (0.710, 1.720), *p* = 0.659	N/A	N/A	N/A	N/A	N/A	N/A
	
Multiple Suicide Attempts vs Single	N/A	N/A	N/A	OR 0.896, 95% CI (0.481, 1.670), *p* = 0.730	OR 0.841, 95% CI (0.445, 1.590), *p* = 0.595	OR 0.842, 95% CI (0.445, 1.595), *p* = 0.598	N/A	N/A	N/A
	
BSS Score Rank Transformed	*F*_(1,400)_ = 2.139, *p* = 0.144	*F*_(1,399)_ = 3.377, p = 0.067* ↑	*F*_(1,397)_ = 3.081, *p* = 0.080* ↑	*F*_(1,198)_ = 3.973, *p* = 0.048**, *r*^2^ = 0.020 ↑	*F*_(1,197)_ = 5.53, *p* = 0.020**, *r*^2^ = 0.039 ↑	*F*_(1,195)_ = 4.98, *p* = 0.027**, *r*^2^ = 0.048 ↑	*F*_(1,200)_ = 0.682, *p* = 0.410	*F*_(1,199)_ = 0.333, *p* = 0.564	*F*_(1,197)_ = 0.024, *p* = 0.876
	
BIS Total Score Top Quartile	OR 1.557, 95% CI (0.977, 2.480), *p* = 0.063* ↑	OR 1.696, 95% CI (1.052, 2.734), *p* = 0.030** ↑	OR 1.656, 95% CI (1.024, 2.678), *p* = 0.040** ↑	OR 1.779, 95% CI (0.961, 3.292), *p* = 0.067* ↑	OR 1.998, 95% CI (1.056, 3.779), *p* = 0.033** ↑	OR 2.020, 95% CI (1.064, 3.835), *p* = 0.032** ↑	OR 1.240, 95% CI (0.564, 2.727), *p* = 0.593	OR 1.332, 95% CI (0.593, 2.949), *p* = 0.495	OR 1.278, 95% CI (0.568, 2.876), *p* = 0.554
	
BPAQ Physical Aggression Factor Score Total	*F*_(1,403)_ = 0.973, *p* = 0.324	*F*_(1,402)_ = 0.127, *p* = 0.721	*F*_(1,400)_ = 0.000, *p* = 0.989	*F*_(1,199)_ = 0.571, *p* = 0.451	*F*_(1,198)_ = 0.016, *p* = 0.899	*F*_(1,196)_ = 0.33, *p* = 0.565	*F*_(1,202)_ = 0.656, *p* = 0.419	*F*_(1,201)_ = 0.505, p = 0.478	*F*_(1,199)_ = 0.06, *p* = 0.807
	
BPAQ Anger Factor Score Total	*F*_(1,403)_ = 0.110, *p* = 0.741	*F*_(1,402)_ = 0.002, *p* = 0.968	*F*_(1,400)_ = 0.083, *p* = 0.773	*F*_(1,199)_ = 0.187, *p* = 0.666	*F*_(1,198)_ = 0.013, *p* = 0.908	*F*_(1,196)_ = 0.01, *p* = 0.921	*F*_(1,202)_ = 0.036, p = 0.850	*F*_(1,201)_ = 0.071, *p* = 0.790	*F*_(1,199)_ = 0.04, *p* = 0.844
	
BPAQ Verbal Aggression Factor Score Total	*F*_(1,403)_ = 0.008, *p* = 0.928	*F*_(1,402)_ = 0.230, *p* = 0.632	*F*_(1,400)_ = 0.015, *p* = 0.902	*F*_(1,199)_ = 0.831, *p* = 0.363	*F*_(1,198)_ = 0.107, *p* = 0.744	*F*_(1,196)_ = 0.24, *p* = 0.625	*F*_(1,202)_ = 0.967, *p* = 0.327	*F*_(1,201)_ = 1.026, *p* = 0.312	*F*_(1,199)_ = 0.81, *p* = 0.370
	
BPAQ Hostility Factor Score Total	*F*_(1,403)_ = 0.115, *p* = 0.735	*F*_(1,402)_ = 0.361, *p* = 0.548	*F*_(1,400)_ = 0.015, *p* = 0.902	*F*_(1,199)_ = 0.135, *p* = 0.714	*F*_(1,198)_ = 0.653, *p* = 0.420	*F*_(1,196)_ = 1.2, *p* = 0.273	*F*_(1,202)_ = 0.004, *p* = 0.951	*F*_(1,201)_ = 0.004, *p* = 0.947	*F*_(1,202)_ = 0.10, *p* = 0.752
	
BPAQ Score Total	*F*_(1,403)_ = 0.106, *p* = 0.745	*F*_(1,402)_ = 0.026, *p* = 0.873	*F*_(1,400)_ = 0.036, *p* = 0.849	*F*_(1,199)_ = 0.222, *p* = 0.638	*F*_(1,198)_ = 0.083, *p* = 0.774	*F*_(1,196)_ = 0.19, *p* = 0.663	*F*_(1,202)_ = 0.025, *p* = 0.874	*F*_(1,201)_ = 0.020, *p* = 0.886	*F*_(1,199)_ = 0.03, *p* = 0.878
	
Log of BDI Score Total	*F*_(1,360)_ = 1.716, *p* = 0.191	*F*_(1,359)_ = 1.816, *p* = 0.179	*F*_(1,357)_ = 0.380, *p* = 0.538	*F*_(1,185)_ = 4.216, *p* = 0.041**, *r*^2^ = 0.022 ↑	*F*_(1,184)_ = 5.447, *p* = 0.021**, *r*^2^ = 0.035 ↑	*F*_(1,182)_ = 3.136, *p* = 0.078* ↑	*F*_(1,173)_ = 0.107, *p* = 0.744	*F*_(1,172)_ = 0.186, *p* = 0.667	*F*_(1,170)_ = 0.0530, *p* = 0.469
	
Depressed by BDI Score ≥14	OR 1.239, 95% CI (0.800, 1.920), *p* = 0.337	OR 1.280, 95% CI (0.820, 1.998), *p* = 0.277	OR 1.236, 95% CI (0.788, 1.940), *p* = 0.357	OR 1.619, 95% CI (0.830, 3.155), *p* = 0.157	OR 2.016, 95% CI (1.001, 4.059), *p* = 0.050** ↑	OR 2.037, 95% CI (1.007, 4.120), *p* = 0.048** ↑	OR 0.943, 95% CI (0.504, 1.766), *p* = 0.855	OR 0.871, 95% CI (0.460, 1.651), *p* = 0.673	OR 0.781, 95% CI (0.405, 1.507), *p* = 0.462
	
Sleep Disturbance	OR 1.073, 95% CI (0.676, 1.703), *p* = 0.764	OR 0.995, 95% CI (0.621, 1.594), *p* = 0.984	OR 0.973, 95% CI (0.606, 1.561), *p* = 0.909	OR 0.702, 95% CI (0.360, 1.367), *p* = 0.298	OR 0.662, 95% CI (0.335, 1.311), *p* = 0.327	OR 0.664, 95% CI (0.335, 1.316), *p* = 0.241	OR 1.524, 95% CI (0.795, 2.920), *p* = 0.205	OR 1.397, 95% CI (0.721, 2.709), *p* = 0.322	OR 1.345, 95% CI (0.689, 2.624), *p* = 0.385
	
Prolonged Sleep Latency	OR 1.051, 95% CI (0.651, 1.697), *p* = 0.838	OR 1.141, 95% CI (0.699, 1.861), *p* = 0.598	OR 1.133, 95% CI (0.694, 1.850), *p* = 0.618	OR 1.117, 95% CI (0.591, 2.113), *p* = 0.734	OR 1.293, 95% CI (0.669, 2.501), *p* = 0.445	OR 1.294, 95% CI (0.669, 2.503), *p* = 0.444	OR 0.936, 95% CI (0.444, 1.972), *p* = 0.861	OR 0.958, 95% CI (0.450, 2.041), *p* = 0.912	OR 0.951, 95% CI (0.443, 2.040), *p* = 0.897
	
Daytime Dysfunction due to Sleepiness	OR 1.147, 95% CI (0.666, 1.975), *p* = 0.620	OR 1.296, 95% CI (0.743, 2.259), *p* = 0.361	OR 1.261, 95% CI (0.722, 2.204), *p* = 0.415	OR 1.997, 95% CI (0.776, 5.140), *p* = 0.152	OR 2.850, 95% CI (1.059, 7.672), *p* = 0.038** ↑	OR 2.849, 95% CI (1.058, 7.675), *p* = 0.038** ↑	OR 0.791, 95% CI (0.395, 1.580), *p* = 0.506	OR 0.807, 95% CI (0.400, 1.630), *p* = 0.550	OR 0.753, 95% CI (0.369, 1.538), *p* = 0.436
	
Impaired Sleep Efficiency	OR 0.740, 95% CI (0.465, 1.176), *p* = 0.202	OR 0.779, 95% CI (0.487, 1.247), *p* = 0.298	OR 0.783, 95% CI (0.488, 1.254), *p* = 0.309	OR 0.563, 95% CI (0.297, 1.065), *p* = 0.077* ↓	OR 0.603, 95% CI (0.315, 1.154), *p* = 0.127	OR 0.603, 95% CI (0.315, 1.155), *p* = 0.127	OR 0.979, 95% CI (0.496, 1.933), *p* = 0.951	OR 1.015, 95% CI (0.509, 2.023), *p* = 0.966	OR 1.086, 95% CI (0.539, 2.186), *p* = 0.817
	
Impaired Overall Sleep Quality	OR 0.966, 95% CI (0.627, 1.490), *p* = 0.876	OR 1.065, 95% CI (0.684, 1.659), p = 0.780	OR 1.068, 95% CI (0.685, 1.665), *p* = 0.773	OR 0.661, 95% CI (0.359, 1.216), *p* = 0.183	OR 0.733, 95% CI (0.393, 1.368), *p* = 0.329	OR 0.733, 95% CI (0.392, 1.370), *p* = 0.331	OR 1.403, 95% CI (0.754, 2.608), *p* = 0.285	OR 1.541, 95% CI (0.817, 2.909), *p* = 0.182	OR 1.660, 95% CI (0.868, 3.172), *p* = 0.125
	
PSQI Total Score	*F*_(1,397)_ = 0.130, *p* = 0.719	*F*_(1,396)_ = 0.032, *p* = 0.859	*F*_(1,394)_ = 0.034, *p* = 0.854	*F*_(1,195)_ = 1.392, *p* = 0.239	*F*_(1,194)_ = 0.628, *p* = 0.429	*F*_(1,192)_ = 0.311, *p* = 0.578	*F*_(1,200)_ = 0.223, *p* = 0.637	*F*_(1,199)_ = 0.163, *p* = 0.687	*F*_(1,197)_ = 0.408, *p* = 0.524

* indicates statistical trend (0.05 < *p* ≤ 0.1);

** indicates statistical significance (0.01 ≤ *p* ≤ 0.05);

↑ indicates a positive association between the variable and TQ-Tg-IgG (higher in the *T. gondii* top quartile cases);

↓ indicates a negative association between the variable and TQ-Tg-IgG (lower in the *T. gondii* top quartile cases); OR > 1 indicates a positive association (variable is higher in *T. gondii* top quartile of serointensity cases); OR < 1 indicates a negative association (variable is lower in *T. gondii* top quartile of serointensity cases); BDI – Beck Depression Inventory; BIS – Barratt Impulsiveness; BPAQ – Buss-Perry Aggression Questionnaire; BSS – Beck Scale for Suicidal Ideation; N/A – Not Analysable; PSQI – Pittsburgh Sleep Quality Index

**Table 10.** Adjustment for PTSD of the positive significant associations of high versus low serointensity groups (high quartile versus bottom three quartiles) and selective suicide risk factors (suicidal ideation, depression, impulsivity, and daytime dysfunction due to sleepiness) in Veterans with a history of suicide attempt. All associations were adjusted for age

Variable	PTSD current*	PTSD lifetime*	PTSD by PCL-5 (>33)**
Suicidal ideation (BSS Score Rank)	*F*_(1,192)_ = 6.989, *p* = 0.009***, *r*^2^ = 0.084 ↑	*F*_(1,192)_ = 5.208, *p* = 0.024**, *r*^2^ = 0.051 ↑	*F*_(1,195)_ = 4.119, *p* = 0.044**, *r*^2^ = 0.178 ↑
	
Depressed by BDI Score ≥14	OR 2.237, 95% CI (1.064, 4.699), *p* = 0.034** ↑	OR 2.162, 95% CI (1.047, 4.465), *p* = 0.037** ↑	OR 2.625, 95% CI (1.111, 6.202), *p* = 0.028** ↑
	
Log of BDI Score Total	*F*_(1,179)_ = 6.771, *p* = 0.010**, *r*^2^ = 0.111 ↑	*F*_(1,179)_ = 7.019, *p* = 0.009***, *r*^2^ = 0.092 ↑	*F*_(1,182)_ = 9.426, *p* = 0.002***, *r*^2^ = 0.387 ↑
	
BIS Total Score Top Quartile	OR 2.176, 95% CI (1.120, 4.229), *p* = 0.022** ↑	OR 2.141, 95% CI (1.113, 4.120), *p* = 0.023** ↑	OR 2.041, 95% CI (1.052, 3.960), *p* = 0.035** ↑
	
Daytime Dysfunction due to Sleepiness (Categorical)	OR 2.954, 95% CI (1.088, 8.020), *p* = 0.034** ↑	OR 3.075, 95% CI (1.123, 8.417), *p* = 0.029** ↑	OR 2.957, 95% CI (1.064, 8.216), *p* = 0.038** ↑

* By SCID for DSM-5 (Structured Clinical Interview for Diagnostic and Statistical Manual of Mental Disorders, Fifth Edition);

** By PCL-5;

↑ indicates a positive association between the variable and TQ-Tg-IgG (higher in the *T. gondii IgG* top quartile cases);

↓ indicates a negative association between the variable and TQ-Tg-IgG (lower in the *T. gondii* top quartile cases); OR > 1 indicates a positive association (variable is higher in *T. gondii* top quartile of serointensity cases); OR < 1 indicates a negative association (variable is lower in *T. gondii* top quartile of serointensity cases);

* indicates statistical trend (0.05 < *p* ≤ 0.1);

** indicates statistical significance (0.01 ≤ *p* ≤ 0.05);

*** indicates statistical significance (0.001 < *p* < 0.01);

**** indicates statistical significance (*p* ≤ 0.001); BDI – Beck Depression Inventory; BSS – Beck Scale for Suicidal Ideation; PCL-5 – Posttraumatic Stress Disorder Checklist-5; PTSD – Posttraumatic Stress Disorder.

**Table 11.** Comparative analysis of SSDV and suicide risk factors between seropositives in the top quartile and seronegatives in the top quartile

Variable	Seropositives in the Top Quartile	Seronegatives in the Top Quartile	Two Sample T-Test/Chi Square	Adjusted for age
*N*	25	90	N/A	N/A
	
Attempted Suicide *N* (%)	12 (48.00)	48 (53.33)	χ^2^ = 0.223, p = 0.637	OR 0.809, 95% CI (0.333, 1.965), *p* = 0.640
	
Multiple Suicide Attempts *N* (%)	8 (32.00)	26 (28.89)	χ^2^ = 0.723, *p* = 0.395	N/A
	
BSS Score Rank Transformed mean (*S.D.*)	200.85 (97.31)	216.73 (101.61)	*t* = 0.686, *p* = 0.494	*F*_(1,111)_ = 0.455, *p* = 0.501
	
BIS Total Score Top Quartile *N* (%)	7 (28.00)	33 (36.67)	χ^2^ = 0.648, *p* = 0.421	OR 0.676, 95% CI (0.255, 1.791), *p* = 0.431
	
BPAQ Physical Aggression Factor Score Total mean (*S.D.*)	19.96 (6.76)	22.07 (8.44)	*t* = 1.149, *p* = 0.253	*F*_(1,112)_ = 1.225, *p* = 0.271
	
BPAQ Anger Factor Score Total mean (*S.D.*)	15.32 (6.96)	17.49 (7.27)	*t* = 1.332, *p* = 0.185	*F*_(1,112)_ = 1.680, *p* = 0.198
	
BPAQ Verbal Aggression Factor Score Total mean (*S.D.*)	14.04 (4.43)	14.14 (4.70)	*t* = 0.100, *p* = 0.921	*F*_(1,112)_ = 0.003, *p* = 0.953
	
BPAQ Hostility Factor Score Total mean (*S.D.*)	19.52 (7.55)	21.87 (7.69)	*t* = 1.355, *p* = 0.178	*F*_(1,112)_ = 1.740, *p* = 0.190
	
BPAQ Score Total mean (*S.D.*)	68.84 (21.11)	75.57 (23.91)	*t* = 1.275, *p* = 0.205	*F*_(1,112)_ = 1.520, *p* = 0.220
	
Log of BDI Score Total mean (*S.D.*)	2.45 (1.08)	2.82 (0.72)	*t* = 1.917, *p* = 0.058* ↓	*F*_(1,98)_ = 3.724, *p* = 0.057* ↓
	
Depressed by BDI Score ≥14 *N* (%)	11 (44.00)	56 (62.22)	χ^2^ = 2.672, *p* = 0.102	OR 0.107, 95% CI (0.195, 1.173), *p* = 0.107
	
Sleep Disturbance *N* (%)	15 (60.00)	63 (70.00)	χ^2^ = 0.897, *p* = 0.344	OR 0.620, 95% CI (0.243, 1.581), *p* = 0.317
	
Prolonged Sleep Latency *N* (%)	6 (24.00)	27 (30.00)	χ^2^ = 0.344, *p* = 0.557	OR 0.744, 95% CI (0.263, 2.099), *p* = 0.576
	
Daytime Dysfunction due to Sleepiness (Yes/No) *N* (%)	20 (80.00)	73 (81.11)	χ^2^ = 0.016, *p* = 0.901	OR 0.985, 95% CI (0.317, 3.064), *p* = 0.979
	
Impaired Sleep Efficiency *N* (%)	6 (24.00)	29 (32.22)	χ^2^ = 0.625, *p* = 0.429	OR 0.669, 95% CI (0.241, 1.856), *p* = 0.440
	
Impaired Overall Sleep Quality *N* (%)	10 (40.00)	48 (53.33)	χ^2^ = 1.391, *p* = 0.238	OR 0.587, 95% CI (0.238, 1.448), *p* = 0.248
	
PSQI Total Score mean (*S.D.*)	9.68 (4.95)	10.13 (4.16)	*t* = 0.462, *p* = 0.645	*F*_(1,112_) = 0.190, *p* = 0.664

* statistical trend;

↓ indicates that the variable is lower in the *T. gondii* seropositive cases); OR > 1 indicates a positive association (variable is higher in *T. gondii* seropositive cases); OR < 1 indicates a negative association (variable is lower in *T. gondii* seropositive cases); BDI – Beck Depression Inventory; BIS – Barratt Impulsiveness; BPAQ – Buss-Perry Aggression Questionnaire; BSS – Beck Scale for Suicidal Ideation; N/A – Not Analysable; PSQI – Pittsburgh Sleep Quality Index.
